# HDAC1-mediated repression of the retinoic acid-responsive gene *ripply3* promotes second heart field development

**DOI:** 10.1371/journal.pgen.1008165

**Published:** 2019-05-15

**Authors:** Yuntao Charlie Song, Tracy E. Dohn, Ariel B. Rydeen, Alex V. Nechiporuk, Joshua S. Waxman

**Affiliations:** 1 Division of Molecular Cardiovascular Biology, Cincinnati Children’s Hospital Medical Center, Cincinnati, OH, United States of America; 2 Molecular and Developmental Biology Graduate Program, University of Cincinnati, Cincinnati, OH, United States of America; 3 Department of Cell and Developmental Biology, Oregon Health & Science University, Portland, OR, United States of America; 4 Department of Pediatrics, University of Cincinnati College of Medicine, Cincinnati, OH, United States of America; University of Pennsylvania School of Medicine, UNITED STATES

## Abstract

Coordinated transcriptional and epigenetic mechanisms that direct development of the later differentiating second heart field (SHF) progenitors remain largely unknown. Here, we show that a novel zebrafish *histone deacetylase 1* (*hdac1)* mutant allele *cardiac really gone* (*crg*) has a deficit of ventricular cardiomyocytes (VCs) and smooth muscle within the outflow tract (OFT) due to both cell and non-cell autonomous loss in SHF progenitor proliferation. Cyp26-deficient embryos, which have increased retinoic acid (RA) levels, have similar defects in SHF-derived OFT development. We found that *nkx2*.*5*^*+*^ progenitors from Hdac1 and Cyp26-deficient embryos have ectopic expression of *ripply3*, a transcriptional co-repressor of T-box transcription factors that is normally restricted to the posterior pharyngeal endoderm. Furthermore, the *ripply3* expression domain is expanded anteriorly into the posterior *nkx2*.*5*^*+*^ progenitor domain in *crg* mutants. Importantly, excess *ripply3* is sufficient to repress VC development, while genetic depletion of Ripply3 and Tbx1 in *crg* mutants can partially restore VC number. We find that the epigenetic signature at RA response elements (RAREs) that can associate with Hdac1 and RA receptors (RARs) becomes indicative of transcriptional activation in *crg* mutants. Our study highlights that transcriptional repression via the epigenetic regulator Hdac1 facilitates OFT development through directly preventing expression of the RA-responsive gene *ripply3* within SHF progenitors.

## Author summary

Congenital heart defects are the most common malformations found in newborns, with many of these defects disrupting development of the outflow tract, the structure where blood is expelled from the heart. Despite their frequency, we do not have a grasp of the molecular and genetic mechanisms that underlie most congenital heart defects. Here, we show that zebrafish embryos containing a mutation in a gene called *histone deacetylase 1* (*hdac1*) have smaller hearts with a reduction in the size of the ventricle and outflow tract. Hdac1 proteins limit accessibility to DNA and repress gene expression. We find that loss of Hdac1 in zebrafish embryos leads to increased expression of genes that are also induced by excess retinoic acid, a teratogen that induces similar outflow tract defects. Genetic loss-of-function studies support that ectopic expression of *ripply3*, a common target of both Hdac1 and retinoic acid signaling that is normally restricted to a subset of posterior pharyngeal cells, contributes to the smaller hearts found in zebrafish *hdac1* mutants. Our study establishes a mechanism whereby the coordinated repression of genes downstream of Hdac1 and retinoic acid signaling is necessary for normal vertebrate outflow tract development.

## Introduction

The ultimate size of all vertebrate hearts is determined through a continuous contribution of differentiating cardiac progenitors, which are often described as the first and second heart field. Earlier-differentiating cardiac progenitors from the first heart field (FHF) migrate to the midline from the anterior lateral plate mesoderm, begin to differentiate into cardiomyocytes (CMs), and form the heart tube. Subsequently, later-differentiating cardiac progenitors from the second heart field (SHF), which lie in the adjacent dorsal and medial pharyngeal mesoderm, contribute to the growth of the heart through adding CMs, as well as smooth muscle and endothelial cells, to the poles [1–9]. In the 4-chambered heart of birds and mammals, the later differentiating cells of the SHF predominantly contribute to both of the atria, the right ventricle, and smooth muscle and endothelial cells of the outflow tract (OFT) [10]. In the comparatively simple two-chamber zebrafish heart, the SHF contributes to the OFT, which includes CMs at the arterial pole of the single ventricle as well as smooth muscle and endothelial cells within the bulbous arteriosus, and a small percentage of cells at the venous pole of the atrium [11–14]. Importantly, despite the differences in chamber number between fish, birds and mammals, conserved signals and transcription factors, including Bmp [15], Wnt [16, 17], Hedgehog (HH) [18, 19], Fgf [20, 21], and retinoic acid (RA) [22–24], regulate the allocation and deployment of cells from the SHF. Although we have gained insight into the roles of these molecular players in guiding SHF development, epigenetic mechanisms that influence their transcriptional inputs during SHF development remain poorly understood.

Significant epigenetic regulation that effects transcription is through modification of histones [25]. One group of these epigenetic modifiers is histone deacetylases (HDACs). Class I HDACs, consisting of HDACs1-3 and HDAC8, are primarily thought to be transcriptional co-repressors, which deacetylate histone tails and consequently causes compaction of chromatin [26]. Despite HDACs of this class being ubiquitously expressed during development, they have been shown to have tissue-specific requirements in development and disease [27]. For instance, while global HDAC1 knockout (KO) mice are early embryonic lethal, largely due to broad proliferation defects [28], tissue-specific deletion within CMs does not produce overt defects, which is thought to be due to redundancy with the nearly identical HDAC2. The requirement of HDAC2 alone may be somewhat controversial. One report indicates *HDAC2* KO mice have no overt defects [29]. However, another study indicated that *HDAC2* KO mice die shortly after birth with enlarged hearts from increased CM proliferation due to hyperacetylation directly on the transcription factor Gata4 [30]. Despite the controversy over HDAC2 alone, functions of HDAC1 and HDAC2 do appear to be redundant in the mammalian heart. Conditional double HDAC1 and HDAC2 KO mice in the heart using the *αMHC*:*Cre* transgene display postnatal defects in cardiac morphogenesis, growth, and contractility that contribute to cardiac arrhythmias and severe ventricular dilation [29]. Unlike mammals, zebrafish do not have a Hdac2 ortholog, suggesting that in zebrafish Hdac1 may perform functions of both Hdac1 and Hdac2 in mammals. Although a previous study suggested that zebrafish *hdac1* mutants may have CM differentiation defects [31], the mechanisms underlying this defect are not understood. Therefore, if HDAC1 homologs regulate aspects of early vertebrate heart development, and specifically SHF development, remains unknown.

HDACs are unable to bind to DNA directly [27]. Thus, their function as epigenetic regulators of transcriptional repression depends on interactions with transcription factors and repressive complexes [27]. Retinoic acid receptors (RARs) are one of the transcription factors involved in recruiting HDACs to DNA [32–37]. At the transcriptional level, the prevailing model is that in the absence of RA the RARs repress transcription of their targets at RA response elements (RAREs) through interaction with transcriptional co-repressors, including HDAC1 [33, 34, 38, 39]. When RA binds to RARs, it induces conformational changes that shed HDACs in favor of transcriptional activators [32, 40, 41]. RA is a potent teratogen [42], with excess embryonic RA signaling perturbing numerous aspects of heart development [43, 44], including preventing SHF accrual to the OFT [24, 45]. Despite this prevailing transcriptional model and recent examples demonstrating alternative mechanisms of ligand-mediate transcriptional repression in the somites [46], there are few examples of requisite RAR-mediated transcriptional repression during development and none that are known to regulate heart development.

In this study, we demonstrate that the zebrafish mutant *cardiac really gone* (*crg)* is a novel *hdac1* loss-of-function allele. Interestingly, *crg* mutants have smaller hearts due to a loss of SHF-derived ventricular cardiomyocytes (VCs) and smooth muscle within the OFT, which is similar to embryos deficient for the RA-degrading Cyp26 enzymes [24]. We identified that *nkx2*.*5*^*+*^ cells from both Hdac1 and Cyp26-deficient embryos have ectopic expression of *ripply3*, a transcriptional co-repressor of T-box (Tbx) transcription factors that is ordinarily restricted to the posterior pharyngeal endoderm. We find excess Ripply3 is sufficient to reduce VC number, while depletion of Ripply3 or its binding partner Tbx1 partially restores VC number in *crg* mutants. Furthermore, consistent with the ectopic expression of *ripply3*, epigenetic markers within the *ripply3* promoter are switched to a state that favors transcriptional activation in *crg* mutants. Altogether, our study reveals that the epigenetic regulator Hdac1 facilitates OFT development through limiting the anterior encroachment of the RA-responsive gene *ripply3* into posterior SHF progenitors.

## Results

### *Crg* mutants display defects in early SHF development

**Fig 1:**
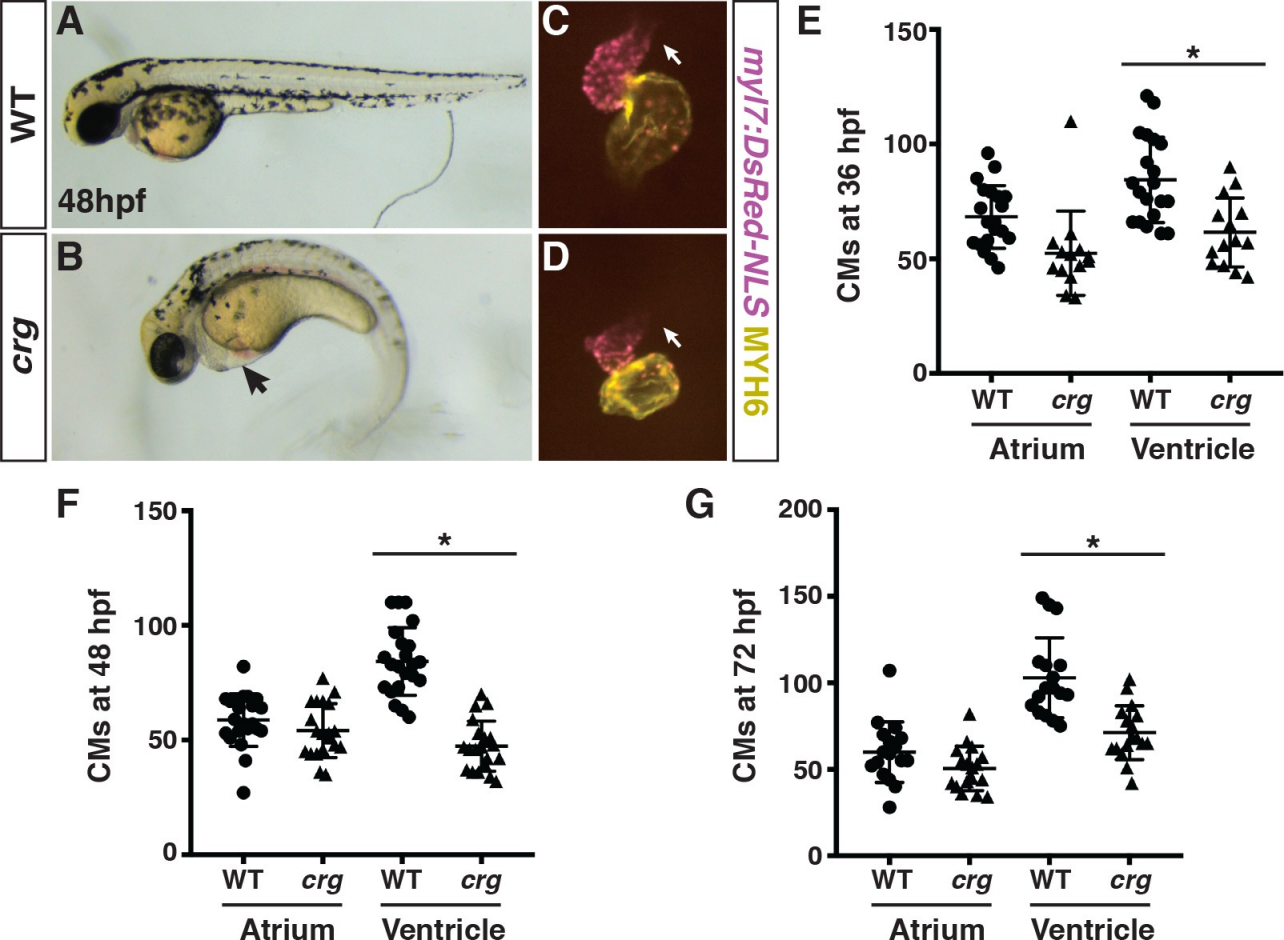
*Crg* mutants have a specific deficit in VCs. (A-B) WT sibling and *crg* mutants at 48 hpf. Lateral views with anterior to the left. Arrow in B indicates pericardial edema. (C-D) Hearts from WT sibling and *crg* mutant *myl7*:*NLS-DsRed2* embryos at 48 hpf. Frontal views. Purple alone indicates ventricle. Yellow indicates atrium. Arrows indicate arterial pole of the ventricle. (E-G) Quantification of CMs in the atria and ventricles of WT sibling and *crg* mutant *myl7*:*DsRed2-NLS* embryos at 36, 48, and 72 hpf. For 36 hpf, n = 14 for WT and *crg* mutants. For 48 hpf, n = 20 for WT and *crg* mutants. For 72 hpf, n = 17 for WT and *crg* mutants. Asterisk in all graphs indicates p<0.05 as determined by Student’s t-test. Error bars for all graphs indicate s.e.m.

Through a N-ethyl N-nitrososurea (ENU)-induced mutagenesis screen for recessive alleles [47], we identified a mutant we named *cardiac really gone* (*crg)* that has overtly smaller hearts coupled with pericardial edema, as well as developmental defects including reduced pigmentation, shortened yolk extension, and a curved body axis (Fig 1A and 1B). Examining hearts with immunohistochemistry (IHC) revealed that in *crg* mutants the hearts are more linear, the size of the ventricle is reduced, and the atria are more bulbous at 48 hours post-fertilization (hpf) compared to wild-type (WT) sibling embryos (Fig 1C and 1D). Interestingly, counting CMs using the *myl7*:*DsRed-NLS* transgene [48] revealed that the number of VCs was reduced in *crg* mutants compared to their WT sibling embryos at 36 hpf through 72 hpf (Fig 1E–1G). However, despite the smaller, bulbous morphology of the atria, the number of atrial cardiomyocytes (ACs) was not significantly affected in *crg* mutants (Fig 1E–1G). Therefore, *crg* mutants have a specific deficit in VCs within the developing hearts of *crg* mutants, which is not caused by developmental delay.

**Fig 2:**
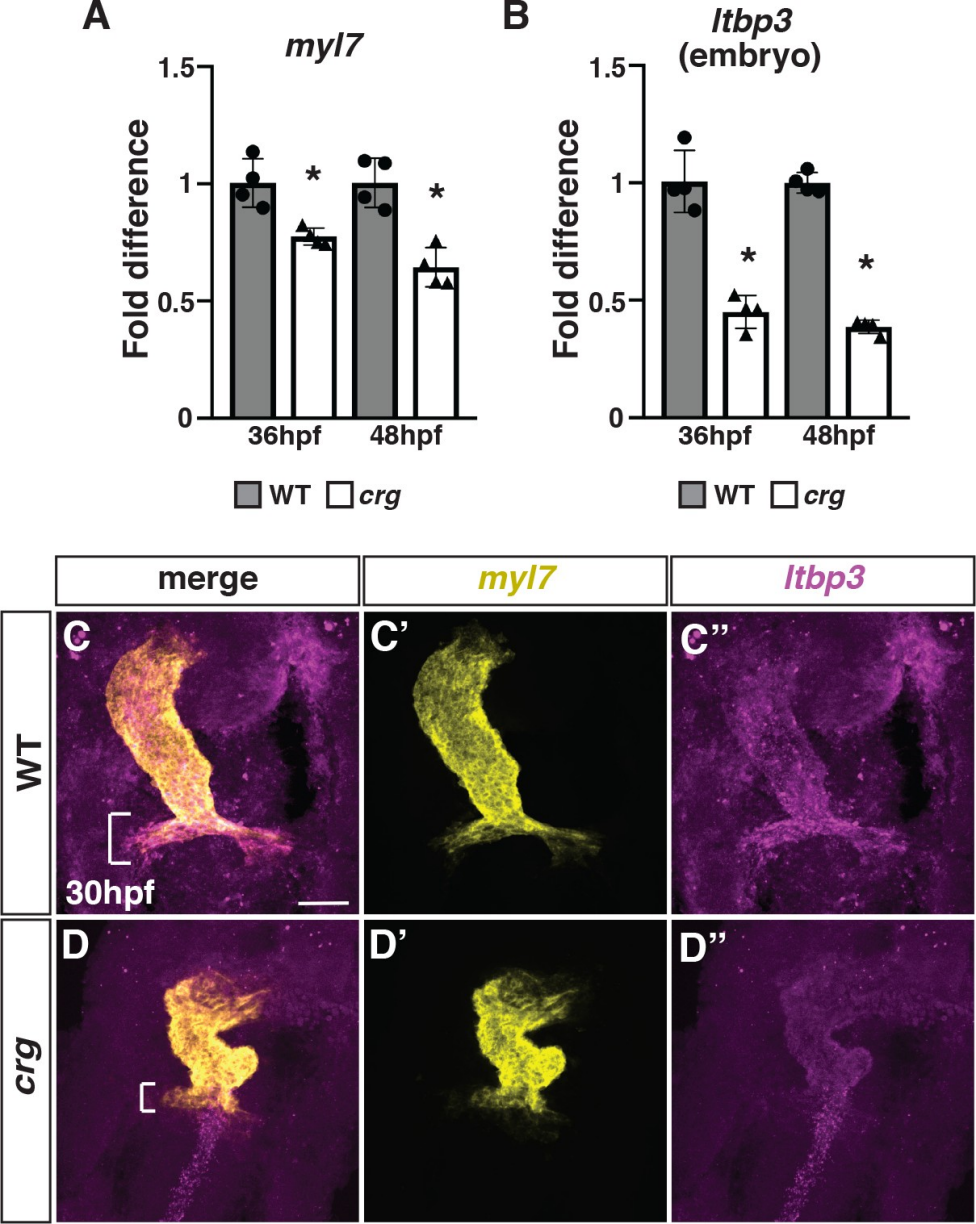
SHF markers of the arterial pole are reduced in *crg* mutants. (A,B) RT-qPCR for the pan-cardiac differentiation marker *myl7* and SHF marker *ltbp3* from embryos at 36 and 48 hpf. (C-D”) Two-color FISH for *myl7* and *ltbp3* in WT sibling and *crg* mutant embryos at 30 hpf. Brackets in C and D indicate presence and absence of *ltbp3* at the arterial pole of WT and *crg* mutant hearts, respectively. n = 5 WT and n = 5 *crg* mutants embryos were examined.

To determine when the heart defects in *crg* mutants first arise, we examined the expression of CM specification and differentiation at earlier embryonic stages. By *in situ* hybridization (ISH), we found that in *crg* mutants neither specification of cardiac progenitors (indicated by *hand2* and *gata4*) was affected at the 8 somite (s) stage nor was early differentiation of CMs (indicated by *myl7*) affected at the 20s stage (S1 Fig), suggesting early cardiac specification and FHF development are not dramatically affected. Therefore, we next quantified expression of *myl7* and the SHF marker *ltbp3* [1] at later stages of heart development with reverse transcription quantitative PCR (RT-qPCR). We found that at both 36 and 48 hpf *myl7* and *ltbp3* expression were reduced (Fig 2A and 2B). Moreover, *ltbp3* expression was absent from the developing OFT of *crg* mutants at 30 hpf using two-color fluorescent *in situ* hybridization (FISH) (Fig 2C–2D”). In contrast to *ltbp3*, we found that the SHF marker *mef2cb* appeared to have increased expression in progenitor cells adjacent to the arterial pole in *crg* mutants at 30 hpf (S2 Fig), although quantitatively we did not find a difference in its expression at 36 hpf in *crg* mutants compared to WT siblings (S2 Fig). Together, these results imply that later-differentiating SHF-derived populations may be lost in *crg* mutants.

**Fig 3:**
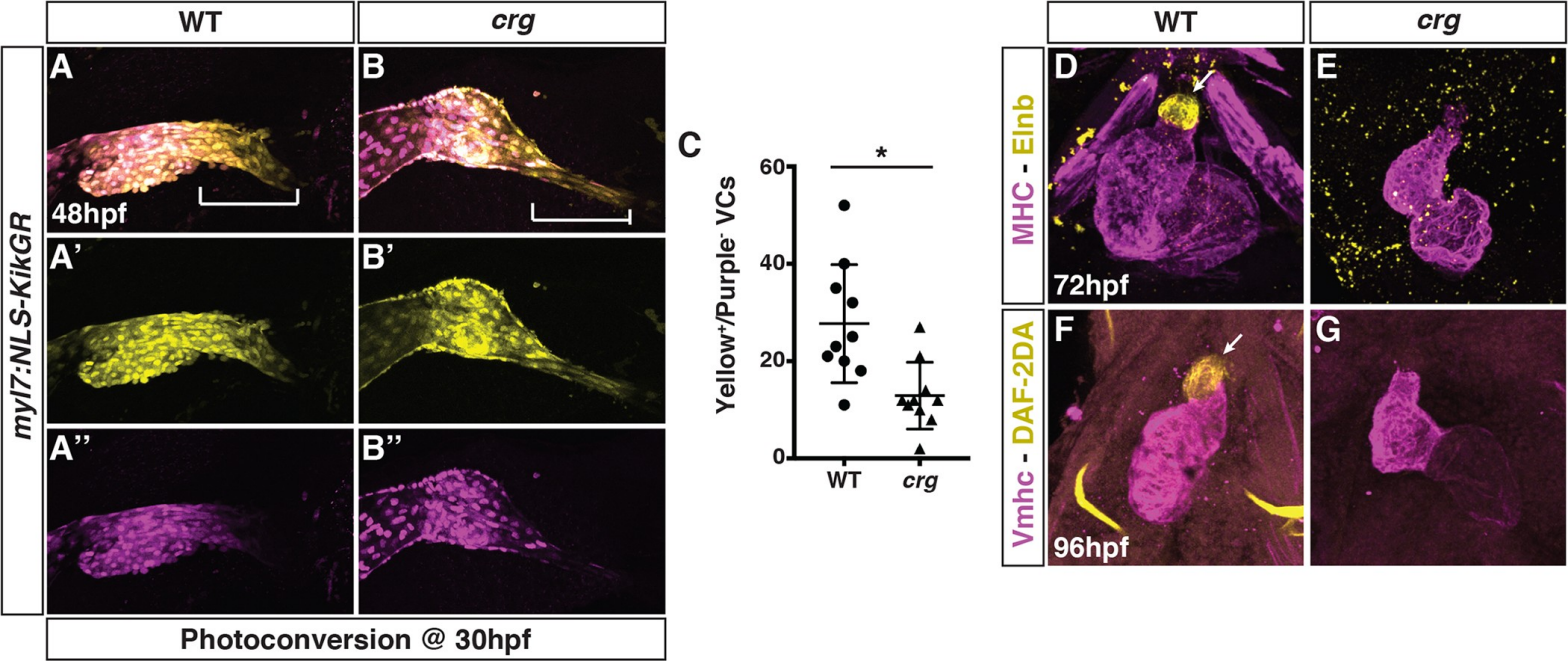
SHF-derived VC and smooth muscle development is impaired in the *crg* mutants. (A-B”) Representative images of hearts from photoconverted WT sibling and *crg* mutant *myl7*:*NLS-KikGR* embryos at 48 hpf. The arterial poles (brackets) are to the right. (C) Quantification of later-differentiating VCs (Yellow^+^/Purple^-^ cells) (n = 10 for WT and *crg* mutants). (D,E) Confocal images of IHC for MHC and Elnb in WT and *crg* mutant embryos at 72 hpf. n = 10 WT and n = 10 *crg* mutants embryos examined. (F,G) Confocal images of DAF-2DA staining coupled with IHC for Vmhc in WT sibling and *crg* mutant embryos at 96 hpf. n = 10 WT and n = 10 *crg* mutants embryos examined. Images in D-G are frontal views with anterior up. Arrows indicate Elnb and DAF-2DA staining of the bulbous arteriosus.

To directly quantify the number of later-differentiating VCs in embryos, we used the established *myl7*:*NLS-Kikgr* transgene [2], which expresses a nuclear photo-convertible KikGR protein in differentiated CMs. Indeed, following conversion of cardiac *NLS-KikGR* from green to red (pseudo-colored as yellow and purple) at 30 hpf, we found that there was an ~50% reduction in the number of later-accruing green^+^ (yellow^+^)/red^-^(purple^-^) VCs within the OFT of *crg* mutants (Fig 3A–3C). Similar trends were obtained using the *myl7*:*Kaede* transgene [4] and photoconverting at 36 hpf (S3 Fig). Next, because the SHF gives rise to smooth muscle in addition to VCs, we analyzed smooth muscle within the OFT using IHC for Elastin b (Elnb*)* and MHC (CMs and skeletal muscle). Elnb^+^ cells of the OFT were not found in *crg* mutants at 72 hpf (Fig 3D and 3E). Consistent with the Elnb IHC, *crg* mutant hearts never stained for DAF-2DA (Fig 3F and 3G), which labels functional smooth muscle [49], further supporting these smooth muscle cells fail to differentiate in *crg* mutants. Altogether, these results support that impaired SHF development produces VC and smooth muscle defects with the arterial ventricle and OFT of *crg* mutants.

### *Crg* mutants contain a novel *hdac1* loss-of-function allele

**Fig 4:**
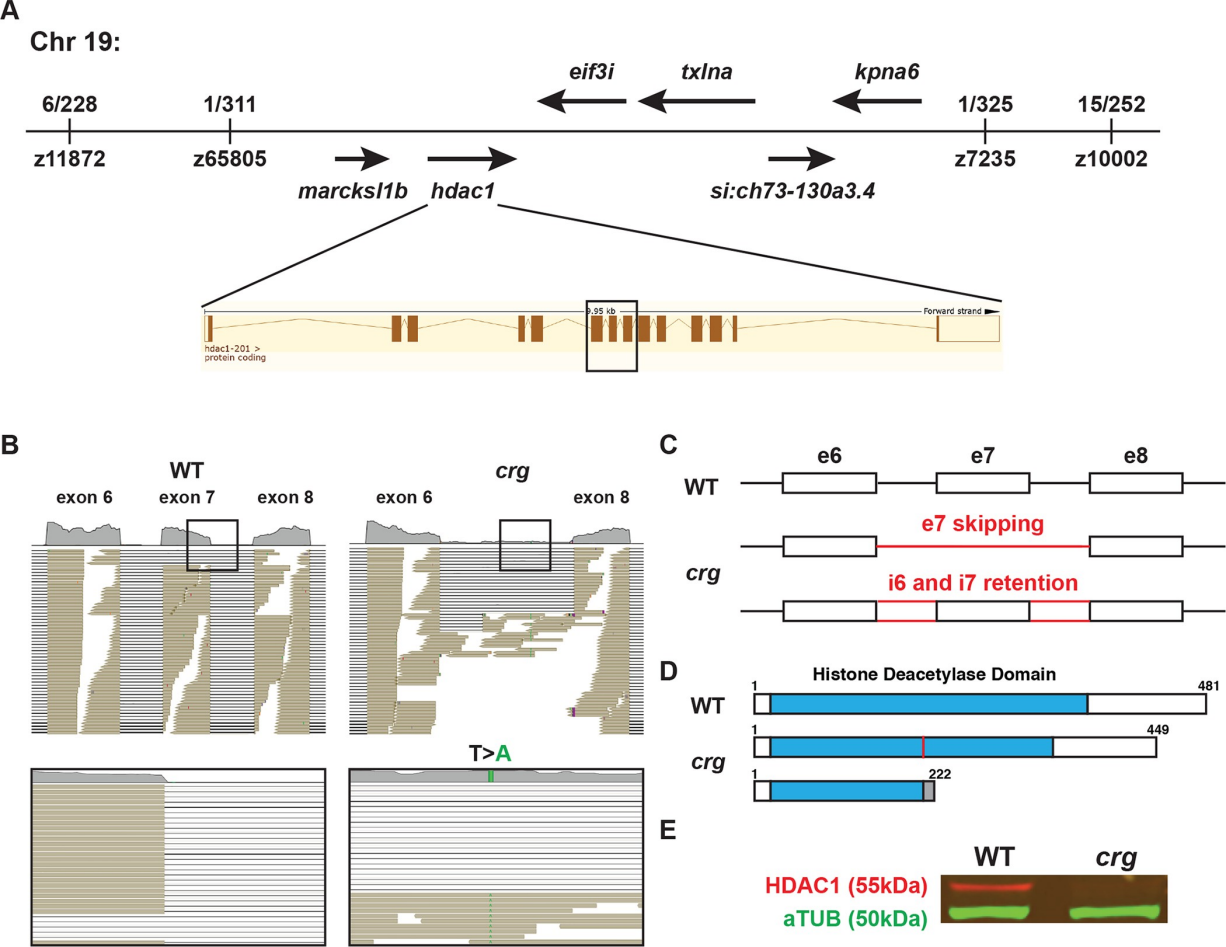
*Crg* mutants contain a loss of function mutation in *hdac1*. (A) Summary of the region containing the *crg* mutation from positional cloning. Fractions indicate recombinants for SSLPs. (B) Reads from RNA-seq data indicating the mutation and effect on the *hdac1* exon 7. Boxes indicate mutation with T to A (green bar) change affecting splice donor site. (C) Schematic indicating the skipping of exon 7 or the retention of intron 6 and 7 found in *hdac1* transcripts from *crg* mutants. (D) Predicted consequences on the proteins generated from the improper splice forms of the *hdac1* transcripts, if they were translated. The red bar indicates the 32 amino acid deletion predicted to occur from the transcript that skips exon 7 in *crg* mutants. The gray bar indicates a 9 amino acid extension after going out of frame at amino acid 213 for the *hdac1* transcript that retains introns 6 and 7 in *crg* mutants. (E) Western blot for Hdac1 protein in WT sibling and *crg* mutants.

To identify the gene affected in *crg* mutants, we conducted traditional positional cloning with simple sequence length polymorphisms (SSLPs), which narrowed down the site of the mutation to within an ~0.5Mb region on Chr19 containing a cluster of 6 genes (Fig 4A). Subsequent RNA-seq on WT sibling and *crg* mutant embryos identified a mutation (T>A) in the splicing donor site of *hdac1’s* exon 7 linked to the *crg* phenotype (Fig 4B). The splice-site mutation appears have two consequences on *hdac1* transcripts: it causes either the skipping of exon 7 or retention of both introns 6 and 7 (Fig 4B and 4C). The two possible outcomes for *hdac1* transcripts in *crg* mutants respectively predicted the potential for proteins to be made with either a 32 amino acid deletion within the histone deacetylase domain or to be severely truncated from going out of frame after amino acid 213 (Fig 4D). However, Western Blot showed that neither WT Hdac1 protein nor smaller predicted proteins were detected in *crg* mutants (Fig 4E and S4 Fig). Thus, our data support that inappropriate splicing of *hdac1* transcripts in the *crg* allele leads to a loss of Hdac1 protein. As further confirmation that the *crg* allele affects *hdac1*, the overt phenotype of *crg* mutants is similar to other previously reported *hdac1* mutant alleles [50–52]. Additionally, injection with a previously verified *hdac1* morpholino oligonucleotide (MO), which inhibits Hdac1 translation (S4 Fig), and treatment with the HDAC inhibitor Trichostatin A (TSA) both produced overt embryo body and cardiac defects equivalent to *crg* mutants (S5 Fig). Importantly, counting the number of CMs in Hdac1-depleted and TSA-treated embryos indicated a specific deficit of VCs in their hearts (S5 Fig), consistent with what we found in *crg* mutants. Therefore, these data show that *crg* is a novel loss-of-function *hdac1* mutant allele and support that Hdac1 loss results in specific deficit of VCs within hearts.

### Hdac1 has cell and cell non-autonomous requirements promoting proliferation of SHF progenitors

**Fig 5:**
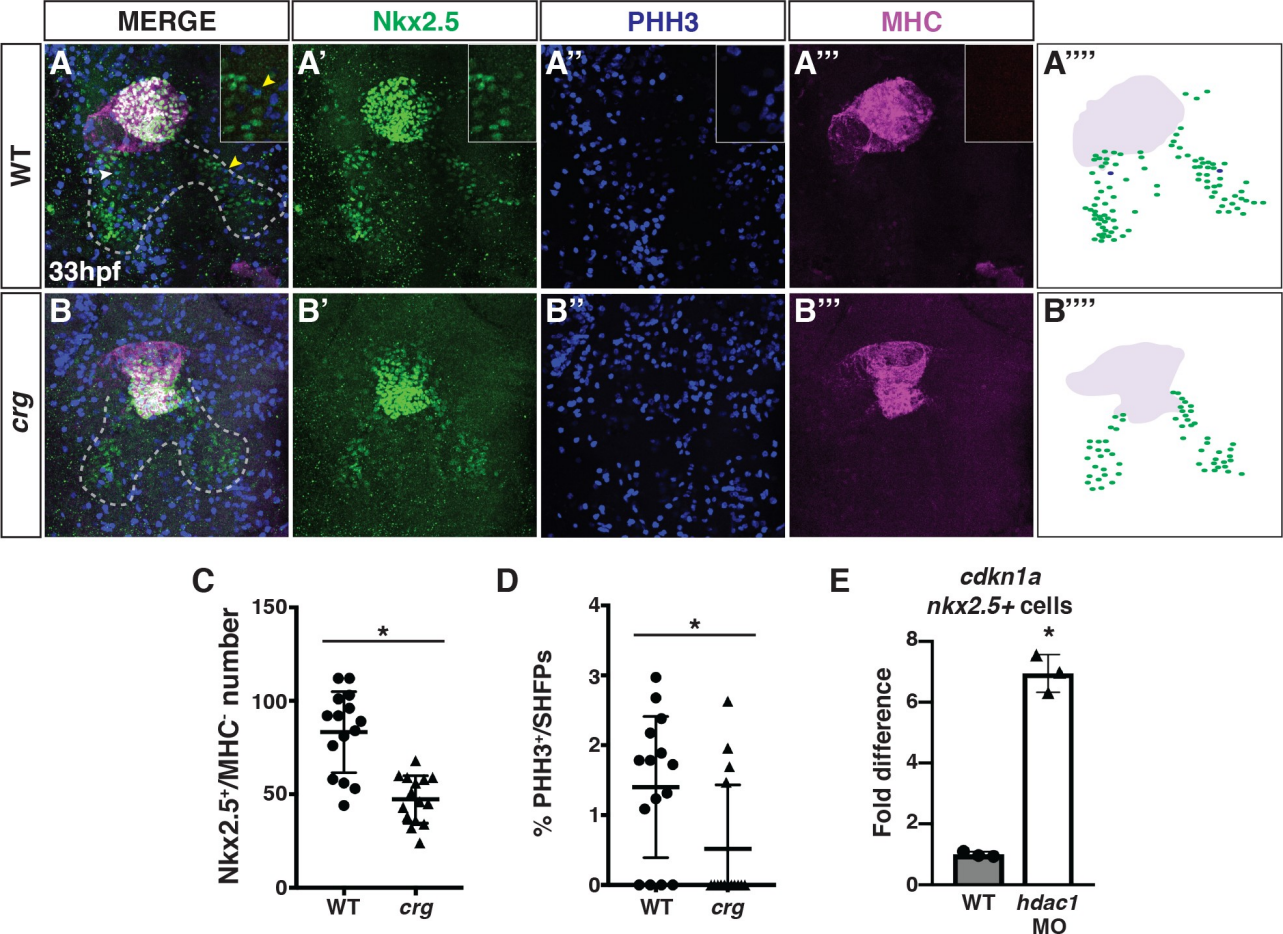
Hdac1 is required for the proliferation of SHF progenitors. (A-B”‘) Confocal images of IHC for hearts and Nkx2.5^+^ SHF progenitors in WT sibling and *crg* mutant embryos at 33 hpf. Nkx2.5^+^ (green), PHH3 (blue) and MHC (purple). Outline in A and B indicates Nkx2.5^+^/MHC^-^ SHF. Arrows indicate Nkx2.5^+^/MHC^-^/PHH3^+^ cells. Yellow arrow indicates Nkx2.5^+^/MHC^-^/pHH3^+^ cell of the higher magnification inset. (A”“and B”“) Schematic indicating IHC from A and B. Green indicates Nkx2.5^+^/MHC^-^ cells. Blue indicates Nkx2.5^+^/MHC^-^/PHH3^+^ cells. Purple indicates MHC^+^ cells. Anterior is up in A-B”“. (C) Quantification of SHF progenitors (Nkx2.5^+^/MHC^-^). (D) Percentage of PHH3^+^ SHF progenitors. For C and D, n = 14 for WT and n = 15 *crg* mutants. (E) RT-qPCR for *cdkn1a* from sorted *nkx2*.*5*:*ZsYellow*^*+*^ cells.

Having found that Hdac1 loss underlies a failure to accrue a significant portion of SHF-derived cells in the OFT of *crg* mutants, we next sought to understand if SHF progenitors were affected in *crg* mutants. We assessed the SHF progenitors using IHC co-staining for Nkx2.5, which marks both SHF progenitors and differentiated CMs, and MHC, which marks differentiated CMs (Fig 5A–5B”). Counting the number of Nkx2.5^+^/MHC^-^ cells adjacent to the OFT at 33 hpf revealed that there are fewer SHF progenitors in *crg* mutants (Fig 5C). Proliferation is reduced in some HDAC1-dependent developmental contexts [28, 53–55]. Therefore, we assessed if reduced cell proliferation may contribute to the diminished number of Nkx2.5^+^ SHF progenitors by co-staining these embryos for the mitotic marker phospho-Histone H3 (PHH3) (Fig 5A–5B”“). Indeed, we found that the percentage of Nkx2.5^+^/MHC^-^/PHH3^+^ cells was reduced in *crg* mutants (Fig 5D). Furthermore, in isolated *nkx2*.*5*:*ZsYellow*^*+*^ cells sorted at 33 hpf, Hdac1-depleted embryos had a dramatic increase in expression of the cell cycle inhibitor *cdkn1a/p21* [56] (Fig 5E). However, while these sorted cells contain SHF progenitors, a caveat is that they likely also contain differentiated cardiomyocytes and pharyngeal arch progenitors [57, 58].

**Fig 6:**
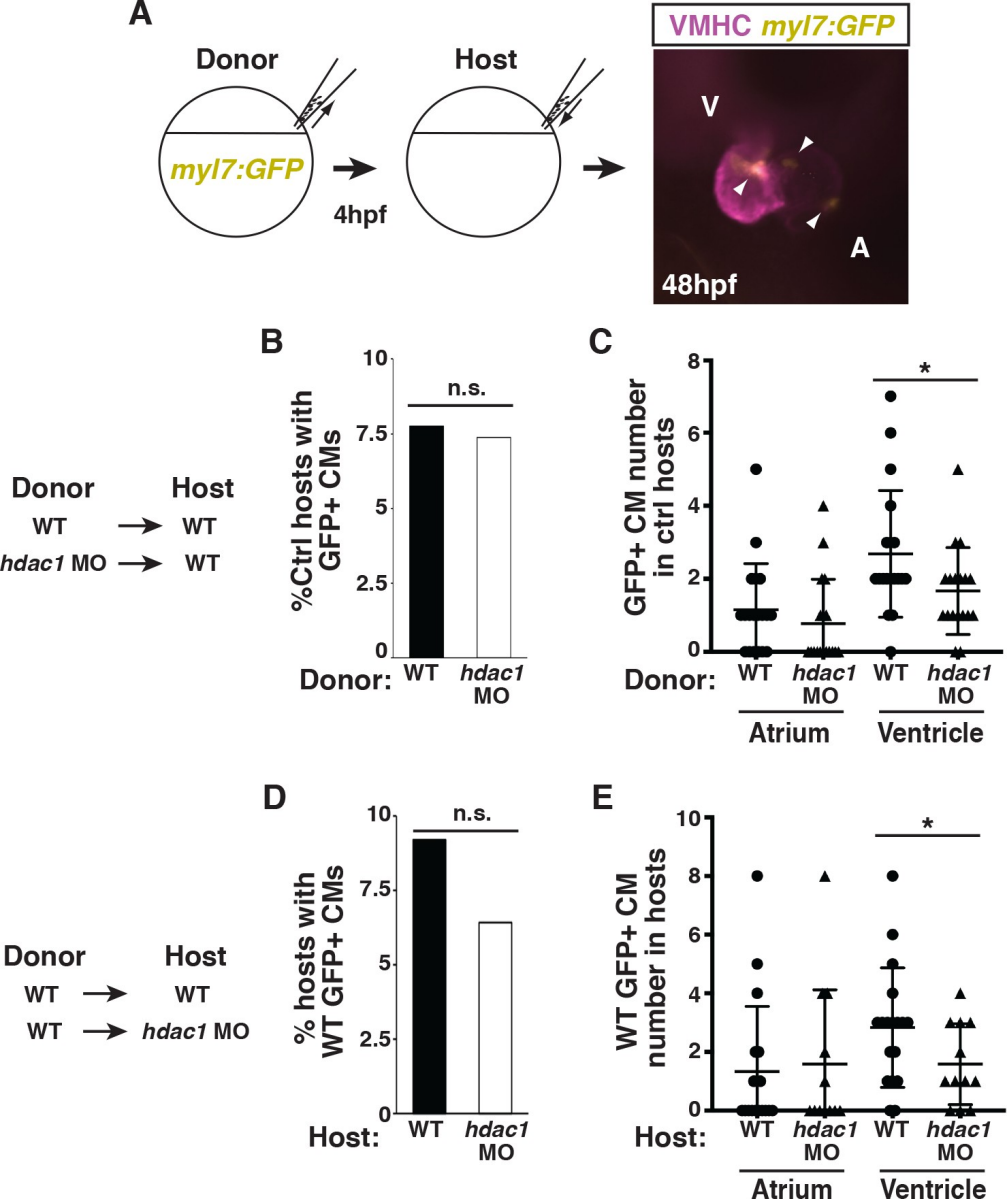
Hdac1 is required both cell and non-cell autonomously to promote VCs. (A) Schematic of the blastula cell transplantation strategy. Vmhc (purple) indicates ventricle. Arrows indicate *myl7*:*GFP*^*+*^ cells (yellow). (B) Frequency of host WT embryos with WT and Hdac1-depleted donor GFP^+^ CMs at 48 hpf. n = 245 WT donor into WT host transplants; n = 244 Hdac1 deficient into WT host transplants. (C) Quantification of donor CMs (GFP^+^) found in host embryos hearts from transplants (n = 19 for WT in WT and n = 18 Hdac1-depleted in WT). (D) Frequency of host WT and Hdac1-depleted embryos with WT donor GFP^+^ CMs at 48 hpf. n = 169 WT donor into WT host transplants; n = 177 WT donor into Hdac1-depleted host transplants. (E) Quantification of donor CMs (GFP^+^) found in host embryos hearts from transplants (n = 18 for WT in WT and n = 12 WT in Hdac1-depleted). Fisher exact test was used to compare significance of frequencies in B and D.

Given *hdac1* is expressed ubiquitously in early zebrafish embryos [52], we next wanted to determine the cells that require Hdac1 for VC development. To assess cell-autonomy, we first performed blastula cell transplantation experiments with donor cells from WT(control) and Hdac1-depleted *myl7*:*GFP* embryos that were placed into WT hosts (Fig 6A). The *myl7*:*GFP* transgene was used so that CMs will unambiguously be marked with GFP in host embryos (Fig 6A). Both the frequency of donor GFP^+^ CM incorporation and the number of donor GFP^+^ CMs in the host atria and ventricles were quantified. When transplanting cells into WT hosts, we found that the frequency of finding donor GFP^+^ CMs was not changed between hosts receiving donor cells from WT or Hdac1-depleted embryos (Fig 6B), suggesting that Hdac1 loss does not affect early CM specification. However, the average number of GFP^+^ CMs found in the ventricles, but not the atria, was significantly reduced from Hdac1-depleted donor cells compared to WT donor cells (Fig 6C). In complementary transplantation experiments where WT donor cells were placed into WT or Hdac1-depleted hosts (Fig 6A), there also was not a statistical change in the frequency of donor GFP^+^ CMs contributing to either set of host embryos (Fig 6D). However, WT donor GFP^+^ CMs demonstrated a decreased frequency of contribution to the ventricles when placed into Hdac1-depleted hosts (Fig 6E). Together, these results are consistent with Hdac1 having both cell-autonomous and cell non-autonomous requirements in promoting the proliferation of SHF progenitors.

### Ripply3 is an effector of Hdac1 in cardiac progenitors

**Fig 7:**
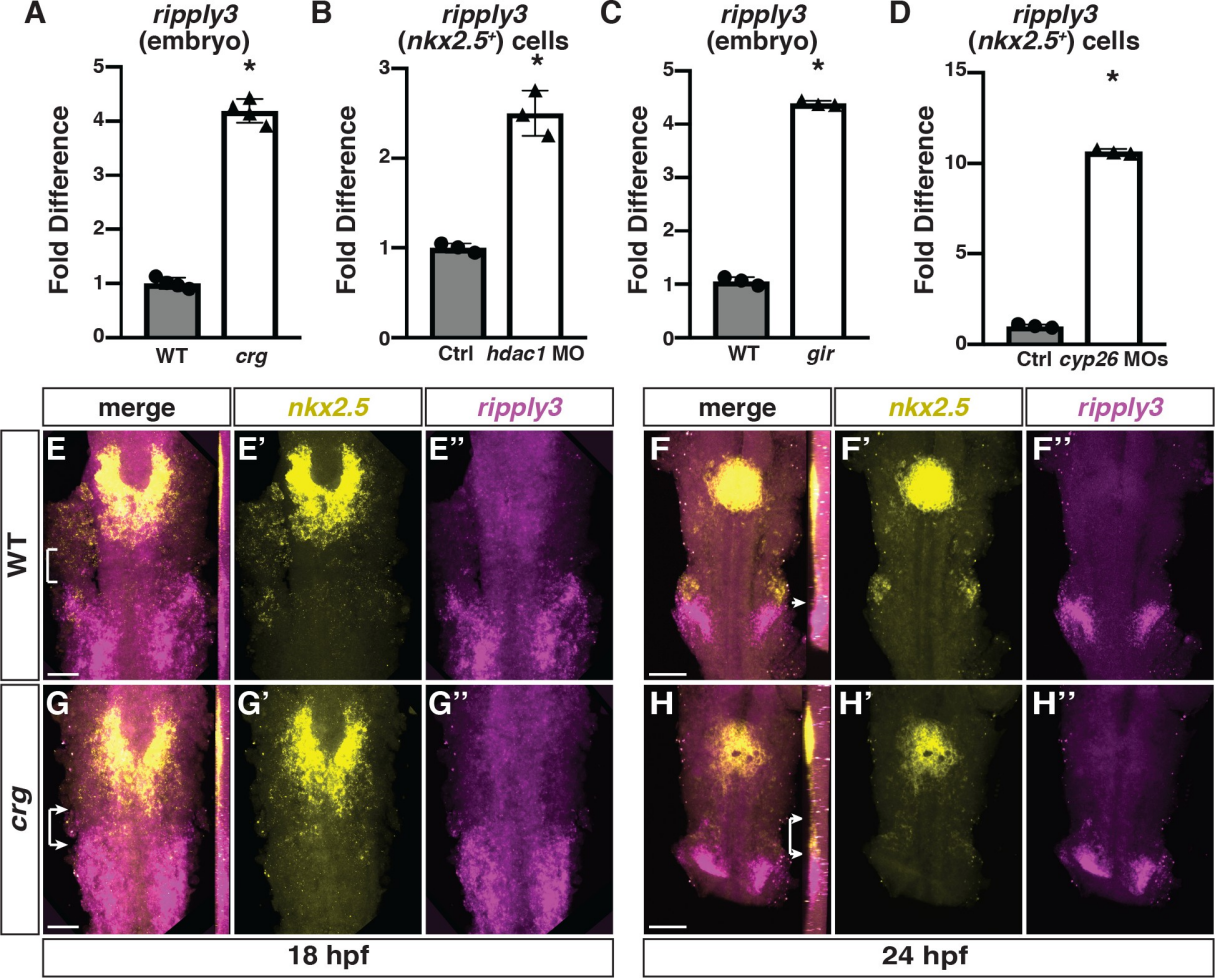
*Ripply3* is expanded anteriorly into *nkx2*.*5+* cells in *crg* mutants. (A-D) RT-qPCR for *ripply3* expression in whole embryos at 48 hpf and sorted *nkx2*.*5*:*ZsYellow+* cells at 28 hpf. (E-H”) Confocal images of two-color FISH for *nkx2*.*5* and *ripply3* in WT and *crg* mutant embryos at 18 and 24 hpf. Images are dorsal views with anterior up. Insets in F-H indicate lateral views of the confocal images. Bracket in E indicates space between posterior *nkx2*.*5* and anterior *ripply3* domains. Arrow in F indicates border between *nkx2*.*5* in pharyngeal mesoderm and *ripply3* in pharyngeal endoderm. Brackets with arrows in G and H indicate overlap in *nkx2*.*5* and *ripply3* domains in *crg* mutant embryos. n = 22 WT and n = 5 *crg* mutants embryos for 18 hpf and n = 19 WT and n = 11 *crg* mutants embryos for 24 hpf examined. Scale bars in E and G are 50 μm. Scale bars in F and H are 100 μm.

RARs are one of the transcription factors that can recruit Hdac1 to chromatin, which it does in the absence of RA ligand [32, 38, 59, 60]. Our recent studies indicate that Cyp26-deficient (loss of both Cyp26a1 and Cyp26c1) embryos, which have ectopic levels of RA, display smaller hearts with a loss of SHF-derived VCs similar to *crg* embryos [24]. Given the established relationship between RARs and Hdac1, we postulated that Hdac1*-* and Cyp26*-*deficiency may result in the de-repression of common effector genes that contribute to SHF-derived OFT defects. To identify candidate genes with increased expression in both Hdac1- and Cyp26*-*deficient embryos, we examined trends of gene expression in RNA-seq analysis of whole embryos at 48 hpf and fluorescence-activated cell sorting (FACS)-isolated *nkx2*.*5*:*ZsYellow*^*+*^ cells at 28 hpf (S6 Fig). One gene we chose for further analysis is *ripply3*, as previous studies in other models have shown its expression is responsive to RA [61, 62]. Therefore, we subsequently confirmed it has increased expression in both Hdac1- and Cyp26-deficient embryos and isolated *nkx2*.*5*:*ZsYellow*^*+*^ cells at these same stages using RT-qPCR (Fig 7A–7D). Additionally, RT-qPCR for SHF marker genes *ltbp3* and *mef2cb* in these isolated *nkx2*.*5*:*ZsYellow*^*+*^ cells showed the same trends that was found when analyzing their expression in mutants (S8 Fig, Fig 2 and S2 Fig)

We were intrigued by *ripply3* (aka *Down syndrome critical region 6* (*DSRC6*)) because it was originally identified as a gene within the portion of chromosome 21 that when supernumerary is associated with Down syndrome [63], which includes a high incidence of congenital heart defects [64]. Mechanistically, previous studies suggest Ripply proteins interact with T-box transcription factors and promote their function as transcriptional repressors [61, 62, 65–67]. Furthermore, *Ripply3* KO mice have OFT and pharyngeal endoderm defects [62], with the latter thought to be from increased expression of Tbx1 target genes. *Ripply3* in mice and Xenopus is restricted to pharyngeal ectoderm and endoderm [61, 67]. Therefore, we examined *ripply3* expression in zebrafish and simultaneously compared its localization to *nkx2*.*5* expression using two-color FISH from the 18s stage through 36 hpf. Similar to *Ripply3* in mice and *Xenopus* [68], we found that by the 18s stage, its expression in zebrafish embryos was bilateral and posterior relative to the majority of *nkx2*.*5* expression in the forming cardiac cone (Fig 7E–7E”). By 24 hpf, *ripply3* resolves to being localized within the posterior pharyngeal endoderm adjacent to *nkx2*.*5* expression within the pharyngeal mesoderm (Fig 7F–7F’), which will give rise to the posterior pharyngeal arch arteries. *Ripply3* maintained this expression in the posterior pharyngeal endoderm adjacent to the developing posterior arch arteries through 36 hpf (S7 Fig).

Intriguingly, at the 18s stage *ripply3* expression was shifted anteriorly in *crg* mutants (Fig 7G–7G”), which is reminiscent to posteriorization of tissues that occurs from excessive RA signaling [69]. Moreover, while the *nkx2*.*5* and *ripply3* expression domains were normally relatively far apart (Fig 7E–7E”), the posterior *nkx2*.*5* and anterior *ripply3* domains overlapped in *crg* mutants at the 18s stage (Fig 7G–7G”). At 24 hpf, *ripply3* expression overlapped with the posterior *nkx2*.*5*^*+*^ cells (Fig 7H–7H”), which at this stage demarcates the developing pharyngeal arch arteries. At subsequent stages, the posterior expression of *nkx2*.*5* was significantly diminished in *crg* mutants compared to WT siblings (S7 Fig). Although we were not able to consistently detect *ripply3* expression within the *nkx2*.*5*^*+*^ domain with FISH at later stages in *crg* mutants (S7 Fig), this is likely due to the lower sensitively of ISH compared to RNA-seq and RT-qPCR. Thus, *ripply3* expression is shifted anteriorly and overlaps with the posterior border of later-differentiating *nkx2*.*5*^*+*^ cardiac cells in *crg* mutants.

**Fig 8:**
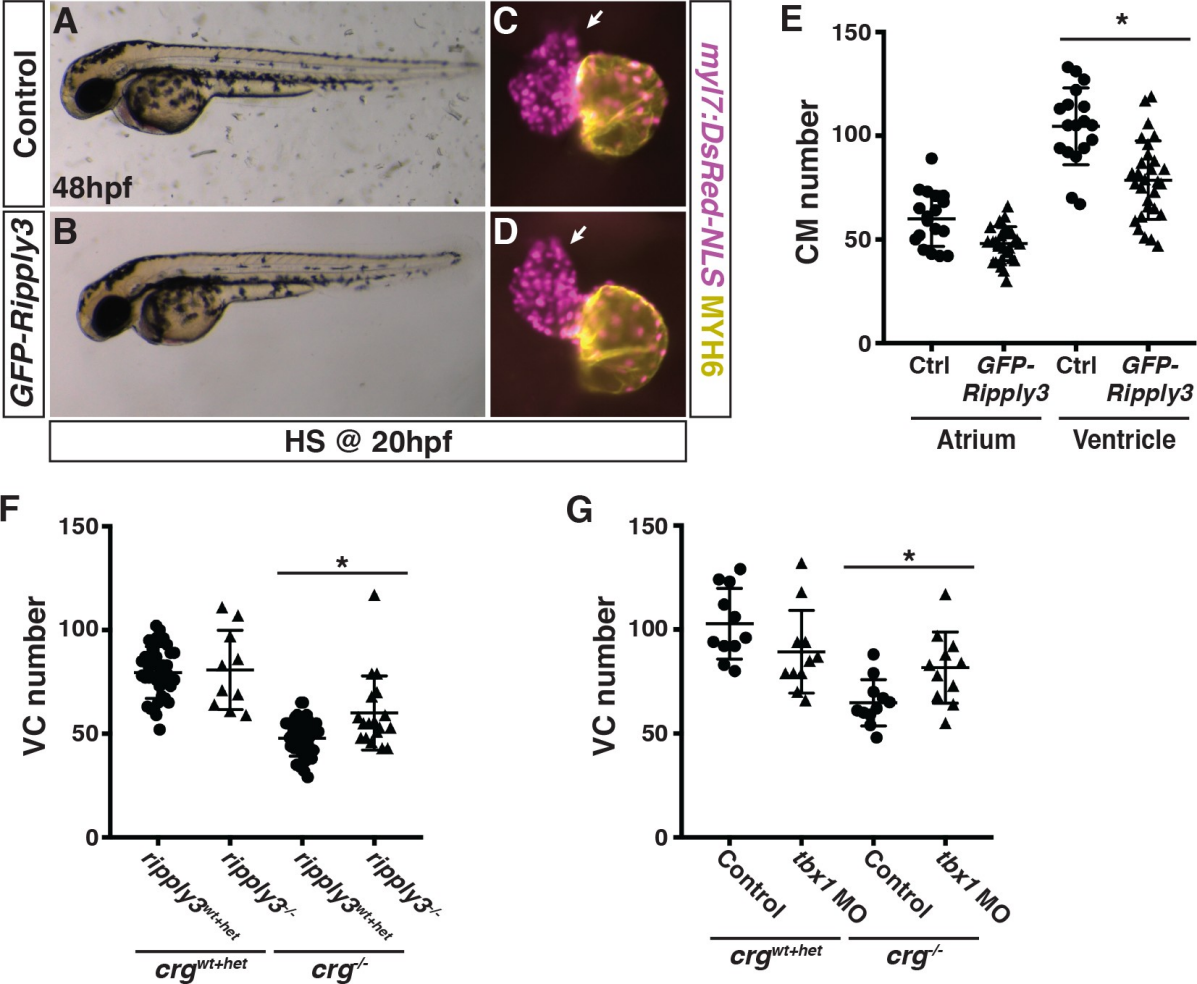
Loss of *ripply3* in *crg* mutants partially restores VC number. (A,B) Control and transgenic *hsp70l*:*GFP-ripply3* embryos at 48 hpf following heat-shock at 20 hpf. (C,D) Hearts from control and *hsp70l*:*GFP-ripply3* transgenic embryos with the *myl7*:*DsRed2-NLS* transgene at 48 hpf following heat-shock at 20 hpf. Purple alone indicates ventricle. Yellow indicates atrium. Arrows indicate arterial pole of the ventricle. (E) Quantification of CMs in control and *hsp70l*:*GFP-ripply3* transgenic embryos at 48 hpf following GFP-*ripply3* induction at 20 hpf (n = 18 for control, n = 29 for *GFP-ripply3*^*+*^). (F) Quantification of VCs in *crg*^*wt+het*^; *ripply3*^*wt+het*^, *crg*^*wt+het*^; *ripply3*^*-/-*^, *crg*^*-/-*^; *ripply3*^*wt+het*^, and *crg*^*-/-*^; *ripply3*^*-/-*^ embryos at 48 hpf (n = 37 for *crg*^*wt+het*^; *ripply3*^*wt+het*^, n = 10 for *crg*^*wt+het*^; *ripply3*^*-/-*^, n = 48 for *crg*^*-/-*^; *ripply3*^*wt+het*^, n = 18 for *crg*^*-/-*^; *ripply3*^*-/-*^). (G) Quantification of VCs in WT (*crg*^*wt+het*^—uninjected) sibling, *crg*^*wt+het*^–Tbx1 depleted, *crg*^*-/-*^*—*uninjected, and *crg*^*-/-*^*—*Tbx1-depleted embryos at 48 hpf (n = 11 for *crg*^*wt+het*^*—*control uninjected, n = 10 for *crg*^*wt+het*^–Tbx1-depleted, n = 10 for *crg*^*-/-*^*—*control uninjected, n = 10 for *crg*^*-/-*^*—*Tbx1-depleted). For all CM quantification, embryos contained the *myl7*:*DsRed2-NLS* transgene.

Because we found ectopic expression of *ripply3* in *crg* mutants, we next determined if excess *ripply3* is sufficient to induce similar VC deficits as *crg* mutants using an inducible transgenic line *Tg(hsp70l*:*GFP-ripply3)* that we generated (S9 Fig). Transgenic *GFP-ripply3* embryos heat-shocked by the tailbud stage (10 hpf) displayed a truncated tail identical to *ripply3* mRNA-injected embryos, supporting that the induced GFP-tagged Ripply3 is functional (S9 Fig). Since our data support that VC deficits in *crg* mutants may arise during the later phase of CM differentiation, we induced *ripply3* expression at the 20s stage, when the heart has started to differentiate and form a cone [70] and approximately when we observed overlap in the cardiac progenitor and *ripply3*^*+*^ fields in *crg* mutants. Although the transgenic embryos were not overtly affected following *GFP-ripply3* induction, we found the hearts were more linear with a reduction in VCs (Fig 8A–8E). Interestingly, *ripply3* mRNA injection or *GFP-ripply3* induction at the tailbud stage also produced a specific reduction in VCs (S9 Fig). Therefore, our data indicate that excess Ripply3 is sufficient to specifically inhibit the development of later-differentiating VCs.

Next, to determine if excess *ripply3* contributes to the VC defects found in *crg* mutants, we engineered *ripply3* mutants using CRISPR-Cas9 [71]. The *ripply3* mutant allele we used deletes 61 bp within the 5’-untranslated region and 1^st^ exon, which encompasses the translational start codon (S10 Fig). This mutation likely leads to non-sense mediated decay or fails to be transcribed, as we could no longer detect expression in the pharyngeal endoderm or via RT-qPCR in *ripply3* mutants (S10 Fig). Unlike the *ripply3* mutant mice, which have cardiac and pharyngeal defects [67], we found that *ripply3* mutant zebrafish had no overt phenotypes and were homozygous viable. However, assessing CM number in *ripply3*; *crg* double mutant embryos, we found that the number of VCs is partially restored in *crg* mutants when *ripply3* is lost (Fig 8F), supporting that excess *ripply3* contributes to the loss of VCs in *crg* mutants. We next assessed if the VC defects in *crg* mutants also require its potential transcriptional partner Tbx1, since Ripply3 promotes transcriptional repression by Tbx1 [72] and Tbx1 has conserved requirements promoting proliferation of SHF progenitors [73–76]. To test this hypothesis, we injected a suboptimal dose of a previously verified *tbx1* MO that phenocopies *van gogh/tbx1* mutants [77] into embryos derived from adult *crg*^*+/-*^ carriers. Use of the suboptimal dose helped overcome recently characterized early specification defects within the anterior lateral plate mesoderm of *van gogh/tbx1* mutants [58]. Embryos were sorted at 48 hpf and the number of VCs quantified, as above. Similar to the *ripply3*; *crg* double mutants, we found a partial restoration of VCs in Tbx1-depleted *crg* mutants (Fig 8G). Manipulation of *ripply3* expression did not affect *tbx1* or *cyp26a1* expression (S11 Fig). Together, these experiments indicate excess *ripply3* contributes to the ventricular OFT defects in *crg* mutants, potentially through promoting transcriptional repression by Tbx1.

### Repression of *ripply3* in *crg* mutants requires RAREs

**Fig 9:**
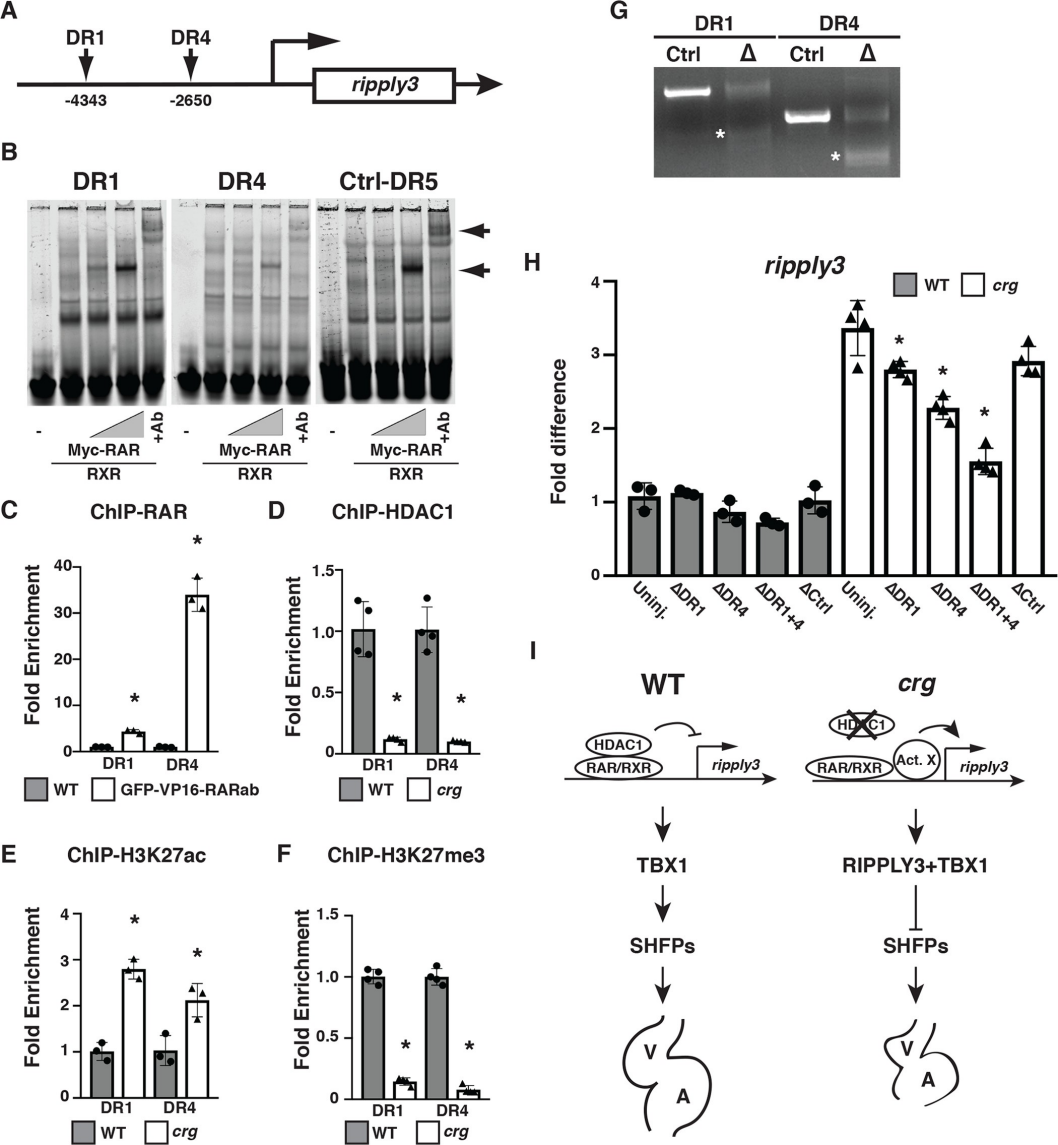
Hdac1 promotes transcriptional repression at RAREs within the *ripply3* promoter. (A) Schematic of DR1 and DR4 RAREs within the *ripply3* promoter. (B) EMSAs with RARs for the *ripply3* DR1 and DR4 sites. The positive control is a DR5 site found in promoters of other direct targets [133]. (C) ChIP-qPCR for induced GFP-VP16-RARab at the DR1 and DR4 sites. (D-F) ChIP-qPCR for HDAC1, H3K27ac, H3K27me3 at the *ripply3* DR1 and DR4 sites in WT sibling and *crg* mutants. Fold enrichment for C-F was normalized versus IgG pull-down. (G) Deletions of the *ripply3* promoter DR1 and DR4 sites using multiplexed gRNAs and Cas9. Asterisks indicate deletions, which were confirmed with Sanger sequencing. (H) RT-qPCR for *ripply3* expression in WT sibling and *crg* mutant embryos with *ripply3* DR1 and DR4 promoter deletions at 36 hpf. The control gRNAs target a region ~100kb away from the *ripply3* promoter. (I) Model depicting Hdac1 and RAR function regulating *ripply3* in SHF development.

We next sought to gain insight into how Hdac1 and RA signaling may be regulating *ripply3* expression. Previous analysis has suggested that RAREs are typically found within 5’kb promoter regions upstream of the transcriptional start site (TSS) of responsive genes [38, 78, 79]. Furthermore, according to the UCSC genome browser (Zv9/danRer7), the 5’kb upstream of the *ripply3* TSS contains epigenetic marks indicative of poised DNA [38, 80–82]. Using NHR scan (www.cisreg.ca) [83] to identify putative RAREs, we found direct repeat 1 (DR1) and DR4 sites within this region (Fig 9A). Although DR4 sites are atypical RAR binding elements [84], electrophoretic mobility shift assays (EMSAs) indicated that RARs can bind both these RAREs *in vitro* (Fig 9B). We were not able to find ChIP-grade antibodies to the endogenous RARs. Therefore, to determine if RARs can bind these sites *in vivo*, we performed ChIP-PCR for GFP-VP16-RARab following heat-shock at 24hpf using our *hsp70l*:*GFP-VP16-RARab* transgenic line [85]. We found the RARs can bind these sites *in vivo* (Fig 9C). Importantly, ChIP-PCR for Hdac1 demonstrated that it no longer associated with these RAREs in *crg* mutants (Fig 9D). To determine if the epigenetic milieu of the *ripply3* promoter was changed in *crg* mutants, we examined the transcriptional activation mark H3K27ac [86] and transcriptional repressive mark H3K27me3 [87] at these RAREs. ChIP-PCR revealed that the RAREs were now associated with transcriptional activation marks and lost transcriptional repression marks in *crg* mutants (Fig 9E and 9F). One possibility for this observation was that ectopic RA directly promoted transcriptional activation of *ripply3* from these cis-regulatory elements. However, in luciferase assays, these elements were not able to activate expression following RA treatment (S12 Fig), instead supporting the idea that RARs may be required to repress *ripply3* expression while some other factor(s) are necessary to activate *ripply3* expression. Consistent with this hypothesis, we found that deleting regions containing either of the RAREs in *crg* mutants using CRISPR-Cas9 led to a loss of ectopic *ripply3* expression at 36 hpf (Fig 9G and 9H), while deletion of both these sites dramatically reduced the ectopic *ripply3* expression (Fig 9H). Injection of control gRNAs to a region ~100kb 3’ to the *ripply3* promoter did not affect expression (Fig 9H). Altogether, these data suggest that HDAC1 promotes a transcriptionally repressive environment within the *ripply3* promoter that prevents other factors from inducing *ripply3* expression.

## Discussion

HDACs control epigenetic modifications to histones that direct gene expression necessary for the proper development of many organs. Previous studies have not illuminated requirements for HDAC1 and/or HDAC2 in SHF development [29, 30]. In contrast to HDAC1 and HDAC2 in mice, deletion of HDAC3 specifically within SHF progenitors using an *Isl1-Cre* results in a spectrum of OFT defects, including double outlet right ventricle and semilunar valve malformations [88]. Despite zebrafish Hdac1 and murine HDAC3 both being Class I HDACs that promote SHF development, it is not clear they are functionally equivalent. OFT defects in HDAC3 SHF KO mice are in part due to increased TGF-β signaling [88, 89]. Additionally, HDAC3 is required for neural crest-derived smooth muscle that septates the distal OFT in mice [89]. In zebrafish, cardiac neural crest cells contribute to ~10% of the cardiomyocytes as well as smooth muscle within the OFT [90–92]. Although zebrafish *hdac1* mutants have impaired neural crest development [93], we have not yet examined the requirement of Hdac1 in cardiac neural crest. Therefore, we cannot rule out that some of the cardiac and smooth muscle deficits we observe are due to requirements within neural crest-derived tissues. However, in contrast to what is observed with SHF-specific HDAC3 KOs in mice, our results suggest that in zebrafish Hdac1 loss impairs SHF progenitor proliferation. Interestingly, TGF-β signaling has been shown to promote proliferation of the SHF progenitors [13], which correlates with the loss of *ltbp3* we observe at the arterial pole of hearts in *crg* mutants. Although global HDAC1-null mice are early embryonic lethal from decreased proliferation affecting many tissues [28] and endodermal progenitors exhibit reduced proliferation in zebrafish *hdac1* mutants [94], loss of proliferation is not a universal consequence of HDAC1. For example, retina defects in zebrafish *hdac1* mutants are due to increased Wnt signaling-dependent proliferation [51]. Therefore, our results showing Hdac1 promotes proliferation of SHF progenitors in zebrafish extends our understanding of the developmental contexts by which this conserved family of epigenetic regulators function in vertebrates.

RARs are one of the transcription factors that recruit HDACs to impart epigenetic modifications and control chromatin accessibility at specific loci [38]. Although proper RA signaling is critical during many stages of heart development, little progress has been made regarding the RA-dependent transcriptional mechanisms used to direct heart development. The canonical model for RAR-mediated transcription implies that RARs can act as transcriptional repressors in the absence of RA and transcriptional activators in the presence of RA [32]. However, recent studies suggest that the RAR transcriptional mechanisms are more versatile. Furthermore, while RARs can complex with HDACs through association with polycomb repressive complex 2 (PRC2) proteins [34, 95, 96], there is limited data suggesting that RARs function as transcriptional repressors in developmental contexts in the absence of ligand [97]. Comparing transcriptomic and ChIP-seq data in cultured cells has indicated that most RAREs are associated with ligand-induced transcriptionally activated targets [38, 98] and that RARs have more flexibility to bind RAREs with highly variable spacing (DR0 through DR8) [98–101]. Our study complements the previous *in vitro* analysis and provides an *in vivo* example that emphasizes the flexibility of RARs as we find they can bind atypical RAREs, in particular at a DR4.

RA-mediated induction of *Ripply3* is likely conserved in vertebrates [61, 62]. Although we still need to decipher the precise transcriptional mechanism by which RA promotes ectopic *ripply3* expression, our results suggest that in contrast to the canonical model of RAR-mediated transcription, RA may not activate *ripply3* expression. Instead, our data provide evidence for a de-repressive transcriptional model whereby excess RA signaling or loss of HDAC1 at these RAREs relieves transcriptional repression of the promoter to allow currently unknown factor(s) to induce ectopic *ripply3* (Fig 9I). In stem cells, unliganded RARs associated with DNA were found to interact with atypical DR0 and DR1 RAREs [98]. While the connection between RA signaling and HDACs has not been reported previously with respect to vertebrate SHF development, it is interesting to consider that both SHF-specific KOs of RARs leads to excess TGF-β that impinges on proper OFT development reminiscent of HDAC3 KOs [88, 102]. Conversely, our results suggest that loss of Hdac1 and excess RA-signaling may impair TGF-β signaling [103]. However, the transcriptional mechanisms by which RA signaling represses TGF-β signaling in mammalian SHF development have not been explored. Therefore, our study reveals insight into the convergence of RAR- and HDAC1-mediated transcriptional repression necessary for SHF development, which may have broad implications for understanding fundamental RA-mediated transcriptional mechanisms.

That *ripply3* expression needs to be restricted to the pharyngeal endoderm for proper OFT development highlights the conserved, intimate relationship between the cardiac and pharyngeal progenitor fields in vertebrates [104–106]. Tbx1 is at the top of a conserved hierarchy of genes that regulates both SHF and pharyngeal development in vertebrates, in part through promoting proliferation and repressing RA signaling [73, 74, 107–110]. Loss of Tbx1 is associated with DiGeorge Syndrome in humans [111], which is characterized by OFT and craniofacial defects. In mice and *Xenopus*, *Ripply3* is restricted to pharyngeal ectoderm and endoderm [67]. Previous work has shown transcriptionally repressive interactions between Ripply3 and Tbx1 are necessary for posterior pharyngeal development [67]. While *Ripply3* KOs also have OFT defects [67], the mechanisms underlying these defects have not been elucidated. Although zebrafish *ripply3* has conserved expression within the posterior pharyngeal endoderm, in contrast to mice, we were surprised to find that *ripply3* is not overtly required for OFT development in zebrafish, or the development of other organs, as they are viable. Thus, the requirement for Ripply3 within the OFT could reflect a function that arose is mammals. Despite the overt lack of requirement in pharyngeal endoderm of zebrafish, our data investigating cardiac defects in *crg* mutants emphasize that it is critical to restrict *ripply3* expression to the posterior endoderm as an expansion of *ripply3* expression into adjacent mesodermal cells inhibits VC production. We propose that SHF defects in *crg* mutants are in part caused by ectopic Ripply3 forcing Tbx1 to function as a transcriptional repressor within these cardiac progenitor cells. As *RIPPLY3 (DSCR6)* is one of the genes in the supernumerary region of chromosome 21 [64], we also speculate its ectopic expression in this context could be one contributing factor to the high incidence of CHDs in children with Down syndrome [112]. Altogether, our data suggest epigenetic repression is necessary to compartmentalize *ripply3* expression to the pharyngeal endoderm as its ectopic expression may impinge on Tbx1 function within SHF progenitors.

Overall, our study reveals insight into how the intersection of HDAC-dependent epigenetic regulation and transcriptional repression by RA signaling promote proper vertebrate OFT development. Because HDAC1 interacts with many other transcription factors and molecular complexes [113], we anticipate that it also regulates SHF development through RA-independent mechanisms. Similarly, we postulate that a combination of direct transcriptional activation and indirect repressive mechanisms, independent of HDAC1 function, contribute to RA-induced teratogenesis and OFT defects. Illuminating how these epigenetic and transcriptional mechanisms are integrated to control gene regulatory networks within SHF progenitors and adjacent organ fields will provide insight into the molecular etiology of developmental syndromes that include congenital heart defects in humans.

## Materials and methods

### Ethics statement

All zebrafish husbandry and experiments were performed as outlined in approved IACUC protocols at the Cincinnati Children’s Hospital Medical Center and Oregon Health and Science University.

### Zebrafish husbandry, transgenic and mutant lines

Adult zebrafish (*Danio rerio*) were raised and maintained under standard laboratory conditions (Westerfield, 2000). Zebrafish transgenic lines used were: *Tg(–5*.*1myl7*:*DsRed-NLS*)^*f2*^ [48], *Tg(hsp70l*:*GFP-VP16-RARab)*^*ci1007*^ [85], *Tg(myl7*:*NLS-KikGR)*^*hsc6*^ *[2]*, *TgBAC(−36nkx2*.*5*:*ZsYellow)*^*fb7*^ *[1]*, *Tg(myl7*:*Kaede)*^*sd22*^ [4], and *Tg(hs70l*:*GFP-Ripply3)*^*ci1011*^. *The Tg(hs70l*:*GFP-Ripply3)* transgenic line used was created using standard Gateway cloning methods and Tol2 mediated transgenesis [114]. The *GFP*-*ripply3* fusion construct was made using PCR. Gateway cloning was used to place the *hsp70l* 5’-entry clone [114], *GFP-Ripply3* middle entry clone, and *polyA* 3’entry plasmids [114] into the *pDEST-Tol2 P2a;α-cry*:*DsRed* plasmid [115]. WT AB/TU embryos were injected with 75 pg of the *hsp70l*:*GFP-ripply3* plasmid and 25 pg *Tol2* mRNA. The F1 progeny of F0 founder fish found to have red eyes were raised. The F1 line selected produced offspring with ~50% containing the transgene, indicative of a single insertion, as well as GFP, and phenotypes equivalent to *ripply3* mRNA injection following heat-shock by the end of gastrulation.

Zebrafish mutant lines used were *ripply3*^*ci1010*^ and *crg*^*nl18*^. *Crg* mutants were identified in an ongoing ENU screen. *Crg* mutants were genotyped using primers *crg-HphI-F2* and *crg-g-R1* (303 bp product) followed by digestion with the restriction enzyme HphI, which cuts the mutant allele. Primers sequences are listed in S1 Table.

### CRISPR-Cas9 generated mutants and deletions

The *ripply3*^*ci1010*^mutant allele was created with CRISPR-Cas9 [71, 116, 117]. Guide RNAs (gRNAs) used were designed using ChopChop (http://chopchop.cbu.uib.no). The gRNA templates were generated using PCR similar to what has been described [71]. The gRNAs were generated using a MEGAshortscript T7 kit (Life Technologies; AM1354). *Ripply3* gRNA (150pg) and *Cas9* RNA (300ng) [118] were injected into embryos at the one-cell stage. Carriers of *ripply3* mutations were identified using PCR on DNA from pooled progeny. Mutations in the F0 and F1 fish were selected for and confirmed through Sanger sequencing. PCR with the primers *ripply3-t1-f1* and *ripply3-t2-r1* were used for genotyping *ripply3* mutants. The WT *ripply3* product is 384 bp and the *ripply3* mutant allele product is 323 bp. Primers sequences used to genotype the *ripply3* mutant are listed in S1 Table.

For deletion of DR sites within the *ripply3* promoter, gRNAs were injected (150 pg/gRNA for a pair; 75 pg/gRNA for the 2 pairs) along with EnGen Cas9 NLS protein (6μM) (New England Biolabs; M0646M). gRNAs and Cas9 protein were diluted to the indicated concentrations in a total volume of 5μl Ultrapure water (ThermoFisher; 1097715). Embryos were injected with 1nl of Cas9/gRNA solution. Co-injection of the gRNAs produced deletions of the predicted size (Fig 9G). Deletion of the target sites with the gRNAs was confirmed by Sanger sequencing. A gRNA pair that targets a genomic region ~100kb 3’ on Chr10 from the *ripply3* promoter was used as a control for RT-qPCR analysis of *ripply3* expression. Primers sequences used to confirm the DR-site deletions and for control gRNAs are listed in S1 Table.

### MO injections

Zebrafish embryos were injected at the one-cell stage with MOs at the following doses: *hdac1* MO 1 ng [52]; *cyp26a1* MO1 2 ng, *cyp26a1* MO2 1 ng, and *cyp26c1* MO 6 ng (injected together) [119]; and *tbx1* MO 1 ng [77]. To counteract non-specific MO-induced cell death, 1 ng *p53* MO was used in all injections [120].

### mRNA synthesis and injection

The coding sequence of *ripply3* was cloned into pCS2p+DEST (a version of the reported pCS2-Dest vector [121] that we modified to have a Pst1 restriction site and corrected T7 primer sequence) using Gateway methods [121]. *Ripply3* mRNA was synthesized using Sp6 Message Machine (Ambion). Embryos were injected with 200 pg *ripply3* mRNA at the one-cell stage.

### TSA treatments

TSA (Sigma; 8552) stock (10 mM DMSO) was diluted to a final concentration of 1 μM in embryo water beginning at 6 hpf. Embryos were incubated until 24 hpf in TSA. Samples were fixed and analyzed by *in situ* hybridization and immunostaining at respective stages.

### Western blotting analysis of protein samples

Western blot assay was performed as described previously [122]. Primary antibodies used were: anti-zebrafish Hdac1 (GeneTex; 124499) and anti-alpha-Tubulin (Sigma; 6199). According to the manufacturer, the anti-zebrafish Hdac1 antibody was generated to amino acids 297–468 of zebrafish Hdac1. Secondary antibodies used were: IRDye 680LT Donkey anti-Rabbit IgG(H+L) (Licor; 92568023) and IRDye 800CW Donkey anti-Mouse IgG(H+L) (Licor; 92532212). Antibodies used are listed on S2 Table.

### ISH and FISH

ISH and two-color FISH were performed as reported previously [123, 124]. Probes used were: *hand2* (ZDB-GENE-000511-1), *gata4* (ZDB-GENE-980526-476), *ltbp3* (ZDB-GENE-060526-130), *mef2cb* (ZDB-GENE-040901-7), *myl7* (formerly called *cmlc2*; ZDB-GENE-991019), *nkx2*.*5* (ZDB-GENE-980526-321), and *ripply3* (ZDB-GENE-060113-3). Plasmid used for *ripply3* probe was a gift of Kazunori Okada and Shinji Takada. For standard ISH, embryos were imaged using a Zeiss M2BioV12 stereomicroscope. For FISH, embryos were imaged using a Nikon A1 inverted confocal microscope. Embryos were genotyped following imaging.

### IHC and CM counting

IHC and counting of cardiomyocytes were performed as previously described [125]. Primary antibodies used were rabbit polyclonal anti-DsRed2 1:1000 (Clontech; 632496), mouse monoclonal anti-Sarcomeric myosin (MHC) 1:10 (MF20; University of Iowa Developmental Studies Hybridoma Bank), mouse monoclonal anti-Atrial myosin heavy chain (AMHC) 1:10 (S46; University of Iowa Developmental Studies Hybridoma Bank), rabbit polyclonal anti-Nkx2.5 1:250 (GeneTex; 128357), mouse monoclonal anti-PHH3 1:500 (Abcam, ab14955), anti-zebrafish Ventricular myosin heavy chain (Vmhc), and anti-zebrafish Elastin b (Elnb). Affinity purified rabbit antibodies to zebrafish Vmhc and Elnb were generated by YenZym (www.YenZym.com) to peptides KSRDVSSKKGHDQE (amino acids 1925–1938) and PGAGYQQQYPGFGGPGAGGPGS (amino acids 1958–1979) of the respective proteins. Secondary antibodies used were goat anti-chicken IgG-FITC (Southern Biotech; 6100–02), goat anti-mouse IgG1-TRITC (Southern Biotech; 1070–02), goat anti-mouse IgG1 FITC (Southern Biotech; 1070–02), goat anti-rabbit IgG-TRITC (Southern Biotech; 4050–03), goat anti-rabbit IgG-FITC (Southern Biotech; 4050–02), goat anti-mouse IgG1-DyLight 405 (BioLegend; Poly24091) and goat anti-mouse IgG2b-TRITC (Southern Biotech; 1090–03) were all used at 1:100. For counting of Nkx2.5^+^ cells and colocalization of Nkx2.5 and PHH3 embryos were imaged using a Nikon A1 confocal microscope. Counting was performed with ImageJ [126]. Colocalization was determined with the aid of Imaris (bitplane.com). All embryos were genotyped by tail clipping. Images were pseudo-colored using ImageJ [126].

### DAF-2DA staining

DAF-2DA was used to stain the smooth muscle of OFT, similar to what has been described [49]. Embryos were transferred to embryo water with 20 γM DAF-2DA (EMD Millipore, 251505-M) at 80 hpf and incubated at 28.5°C in the dark until 96 hpf. Embryos were then harvested and immunostaining was performed as described above with the anti-zebrafish Vmhc antibody to mark the ventricle. The hearts of stained embryos were imaged using a Nikon A1 confocal microscope.

### Photoconversion assays

Photoconversion assays for CM differentiation were performed similar to what has been previously described [24]. Embryos from adult *crg* carrier crosses containing the *myl7*:*NLS-KikGr* or *myl7*:*Kaede* transgenes were exposed to UV at 30 hpf for 30 min on a Zeiss M2BioV12 fluorescent stereomicroscope. Embryos were sorted into WT and *crg* mutant embryos at 48 hpf. Embryos were gently compressed with a footed coverslip and their hearts imaged using a Nikon A1 confocal microscope. ImageJ was used to mark and count the number of nuclei in green-only later differentiating CMs in *myl7*:*NLS-KikGr* transgenic hearts and the area of green-only later differentiating CMs in *myl7*:*Kaede* transgenic hearts. Images were pseudo-colored using ImageJ [126].

### Blastula cell transplantation

To assess cellular autonomy, blastula cell transplantation was performed as previously described [85, 119]. *Tg*(*myl7*:*EGFP*) donor embryos were injected at the one-cell stage with Cascade blue-dextran (Invitrogen) alone or with the *hdac1* MO. At the sphere stage, 15–20 donor cells were transplanted in the margin of WT (control) and/or *hdac1*-deficient host embryos. Host embryos were then grown to 48 hpf and scored for their frequency and contribution to atria and ventricles using a Zeiss M2BioV12 fluorescent stereomicroscope.

### FACS

The preparation of samples from *Tg(nkx2*.*5*:*ZsYellow)* for FACS was performed as described previously [127]. Zombie Aqua (Biolegend) was used to label dead cells. FACS was performed in the CCHMC FACS Core using a BD FACSAria II at 488nm and 561nm. GFP+/Blue- cells were collected for RNA isolation.

### RT-qPCR

cDNA was prepared from whole embryos as previously described [119, 128]. RT-qPCR using SYBR green PCR master mix (Applied Biosystems) was performed under standard PCR conditions in Bio-Rad CFX PCR machine. Relative expression levels of *myl7*, *ltbp3*, *ripply3* and *cdkn1a* were standardized using *β-actin* and the 2^−ΔΔCT^ Livak Method.

### RNA-seq preparation and analysis

Embryos were lysed in Trizol then RNA was isolated using the PureLink RNA Micro Kit (Invitrogen). RNA was submitted to the CCMHC Sequencing core for library preparation. RNA from isolated cells was collected and amplified using a Single Cell RNA purification kit (Norgen Biotek; 51800). RNA was submitted to the CCHMC Gene Expression Core for library preparation. Samples were sequenced in the CCHMC Sequencing core using an Illumina High-Seq 2500 at a depth of >20M reads. Single-end stranded sequencing was performed on whole embryos. Paired-end sequencing was performed on samples from sorted cells. Sequences were aligned to the zebrafish genome (Zv9/danRer7) using Strand-NGS. Differential expression from single conditions of each of the samples was compared using DE-seq. Genes with >2 fold increased expression in Hdac- and Cyp26-deficient conditions relative to control conditions were identified based on manual annotation and clustering of genes with similar expression profiles using the Gene Expression tool kit in Strand NGS. P-values for individual gene expression were not considered for the analysis as they are not informative for statistical analysis of single conditions. The sequencing data have been deposited in GEO (accession # GSE126747).

### EMSA

EMSAs were performed as described previously [129]. Oligonucleotides were designed containing the *ripply3* DR1 site (AGGTCAGAGGTCA), the *ripply3* DR4 (AGTTCCTCAGGGGTCA) site, and a previously reported Cyp26a1 DR5 site [130]. A complementary oligonucleotide was designed with a 5’ LI-COR IRDye 700 (IDT). Sequences for oligos are listed in S1 Table. The oligonucleotides were annealed and the ends filled with Klenow (New England Biolabs). Zebrafish *myc-rarab* was cloned into pCS2+MT. Zebrafish RXRba was cloned into pCS2p+. Proteins for EMSA were made using the TnT SP6 Quick Coupled Transcription/Translation System (Promega). Protein samples were gently mixed with LI-COR tagged probes and incubated at room temperature for 20 minutes. 4% polyacrylamide gels were run for 2 hours at 150 V. Gels were imaged using an Odyssey CLx LI-COR imager.

### ChIP

ChIP was conducted essentially as previously described with 150 embryos per condition [131]. Antibodies used for IPs were: anti-GFP (Abcam; ab290), HDAC1 (Abcam; ab41407), H3K27ac (Abcam; ab4729), and H3K27me3 (Millipore; 7449). All IPs were performed using 1:100 dilutions of the antibodies. The IP’d DNA was analyzed with qPCR described above. Fold enrichment of *ripply3*-DR1 and *ripply3*-DR4 were standardized to that of negative control samples (IgG) (Southern Biotech; 617001). Primer sequences for IPs are listed in S1 Table.

### Cell culture and dual luciferase assay

Fragments of the *ripply3* promoter containing the DR1 (-3890 to -4759 upstream of the TSS) and DR4 (-2241 to -2968 upstream of the TSS) sites were inserted into the EcoRV site of pGL4.23 plasmids (Promega). The 12XRARE-tk-pGL3 vector and luciferase assays were reported and performed in HEK293 cells co-transfected with zebrafish RARab as described previously [132]. Primers for cloning the *ripply3* fragments are listed in S1 Table.

### Statistical analysis

All statistical analysis unless indicated was carried out using two-tailed Student's *t*-test with *p*<0.05 considered to be statistically significant.

## Supporting information

S1 Fig CM specification and early differentiation are overtly unaffected in *crg* mutants. (A-D) ISH for CM specification markers *gata4* and *hand2* at the 8s stage. n = 5 WT and n = 5 *crg* mutants embryos examined. (E,F) ISH for the CM differentiation marker *myl7* at the 20s stage. n = 10 WT and n = 5 *crg* mutants embryos examined. Views are dorsal with anterior up.
(TIF)

S2 Fig The SHF marker *mef2cb* has increased expression adjacent to differentiated CMs at the arterial pole of the heart in *crg* mutants. (A-B”) Two-color FISH for *myl7* and *mef2cb* in WT sibling and *crg* mutant embryos at 30 hpf. Brackets in A and B indicate *mef2cb* at the arterial pole of in WT and *crg* mutant hearts, respectively. n = 5 WT and n = 5 *crg* mutants embryos examined. (C) RT-qPCR for the SHF marker *mef2cb* from embryos at 36 hpf.
(TIF)

S3 Fig Later-differentiating VCs are reduced in *crg* mutants. (A-B”) Images of hearts from photoconverted WT sibling and *crg* mutant *myl7*:*Kaede* embryos at 48 hpf following photoconversion at 36 hpf. The arterial poles (brackets) are to the right. (C) Quantification of the area of later-differentiating VCs (Yellow^+^/Purple^-^). (n = 11 for WT and *crg* mutants).
(TIF)

S4 Fig Whole western blot for Hdac1 in *hdac1* MO-depleted and *crg* embryos. Both the *hdac1* MO and *crg* mutants show a loss of the predicted WT Hdac1 proteins (black arrow). We do not observe the smaller protein with a 32 bp deletion predicted from the transcript analysis in RNA-seq, suggesting that this protein is not generated in the *crg* mutants. Since the antibody used for Western was generated to the C-terminal, it would not recognize the severely truncate protein, if that were made in *crg* mutants. However, a lower band (white arrow) recognized by the antibody, which is potentially a smaller Hdac1 isoform that is present in the WT/Ctrl samples, is also lost in the Hdac1-depleted and *crg* mutant embryos.
(TIF)

S5 Fig Hdac1 depletion and treatment with TSA produce similar phenotypes as *crg* mutants. (A,B) Control and Hdac1-depleted embryos at 48 hpf. Lateral views with anterior to the left. (C,D) Hearts from control and Hdac1-depleted *myl7*:*NLS-DsRed2* embryos at 48 hpf. Frontal views. Purple alone indicates ventricle. Yellow indicates atrium. Arrows indicate arterial pole of the ventricle. (E) Quantification of CMs in the atria and ventricles of control and Hdac1-depleted *myl7*:*NLS-DsRed2* embryos at 48 hpf (n = 19 for control and Hdac1-depleted embryos). (F,G) Control and TSA-treated embryos at 48 hpf. Lateral views with anterior to the left. (H,I) Hearts from control and TSA-treated *myl7*:*NLS-DsRed2* embryos at 48 hpf. Frontal views. Purple alone indicates ventricle. Yellow indicates atrium. Arrows indicate arterial pole of the ventricle. (J) Quantification of CMs in the atria and ventricles of control and TSA-treated *myl7*:*NLS-DsRed2* embryos at 48 hpf (n = 14 for both control and TSA-treated embryos).
(TIF)

S6 Fig Differential expression analysis for whole embryos and sorted *Nkx2.5:ZsYellow+* cells. Heat-maps of genes found to have increased expression from RNA-seq of Hdac1- and Cyp26-depleted embryos at 48 hpf and sorted *nkx2*.*5*:*ZsYellow+* cells at 33 hpf. Scale represents fold-change (log_2_) of normalized values after DE-seq analysis in Strand NGS. Genes represented showed similar trend of increased expression.
(TIF)

S7 Fig Expression of *nkx2.5* and *ripply3* in WT and crg mutants. (A-D”) Confocal images of two-color FISH for *nkx2*.*5* and *ripply3* in WT and *crg* mutant embryos at 30 and 36 hpf. Images are dorsal views with anterior up. Insets in A-D indicate lateral views of the confocal images. n = 10 WT and n = 9 *crg* mutants embryos for 30 hpf and n = 4 WT and n = 4 *crg* mutants embryos for 36 hpf examined. Scale bar is 100 μm.
(TIF)

S8 Fig SHF marker gene expression in Hdac1-depleted *nkx2.5:ZsYellow+* cells. (A) RT-qPCR for *ltbp3* from sorted *nkx2*.*5*^*+*^ cells at 28 hpf. (B) RT-qPCR for *mef2cb* from sorted *nkx2*.*5*^*+*^ cells at 28 hpf.
(TIF)

S9 Fig Generation and validation of the heat-shock inducible *GFP-ripply3* transgenic line. (A) Schematic of the heat-shock inducible *hsp70l*:*GFP-ripply3* transgene. (B-E) Heat-shock induction of *GFP-ripply3* at 24 hpf. Control embryos are heat-shocked non-transgenic siblings. (F,H) Control and *hsp70l*:*GFP-ripply3* embryos at 48 hpf following heat-shock at 10 hpf. Lateral views with anterior to the left. (G,I) Hearts from control and *hsp70l*:*GFP-ripply3*; *myl7*:*NLS-DsRed2* embryos at 48 hpf following heat-shock at 10 hpf. Frontal views. Purple alone indicates ventricle. Yellow indicates atrium. Arrows indicate arterial pole of the ventricle. (J) Quantification of CMs from control and *hsp70l*:*GFP-ripply3*; *myl7*:*NLS-DsRed2* embryos at 48 hpf following heat-shock at 10 hpf (n = 25 for control and *GFP-ripply3*^*+*^). (K,M) Control and *ripply3* mRNA-injected embryos at 48 hpf. Lateral views with anterior to the left. (L,N) Hearts from control and *ripply3* mRNA-injected *myl7*:*NLS-DsRed2* embryos at 48 hpf. Frontal views. Purple alone indicates ventricle. Yellow indicates atrium. Arrows indicate arterial pole of the ventricle. (O) Quantification of CMs from control and *ripply3* mRNA-injected *myl7*:*NLS-DsRed2* embryos at 48 hpf (n = 21 for control and *ripply3* mRNA-injected embryos).
(TIF)

S10 Fig Generation of *ripply3* mutants. (A) Schematic of the *ripply3* exons and the gRNA targeting exon of the *ripply3* gene. (B) The *ripply3* mutant allele used deletes 61bp including the start codon. (C,D) WT sibling and *ripply3* mutants at 48 hpf. (E,F) *Ripply3* transcripts are not detectable in *ripply3* mutants at 30 hpf. Arrows indicate posterior pharyngeal region where *ripply3* is expressed in WT embryos. Views are dorsal with anterior up. (G) RT-qPCR for *ripply3* in *ripply3* mutants at 36 hpf indicates the transcripts are essentially undetectable. Primers used do not bind within the deleted region. (H) Quantification of atrial CMs in *crg*^*wt+het*^; *ripply3*^*wt+het*^, *crg*^*wt+het*^; *ripply3*^*-/-*^, *crg*^*-/-*^; *ripply3*^*wt+het*^, and *crg*^*-/-*^; *ripply3*^*-/-*^ embryos at 48 hpf (n = 37 for *crg*^*wt+het*^; *ripply3*^*wt+het*^, n = 10 for *crg*^*wt+het*^; *ripply3*^*-/-*^, n = 48 for *crg*^*-/-*^; *ripply3*^*wt+het*^, n = 18 for *crg*^*-/-*^; *ripply3*^*-/-*^).
(TIF)

S11 Fig Modulation of *ripply3* expression does not affect *tbx1* or *cyp26a1* expression. (A-C) RT-qPCR for *tbx1* and *cyp26a1* from embryos at 24 and 36 hpf following GFP-Ripply3 induction at the 20s stage and *ripply3* mutants.
(TIF)

S12 Fig The DR sites within the *ripply3* promoter do not respond to RA treatment. Dual luciferase assays in HEK293 cells for the *ripply3* DR1 and DR4 sites. pGL3-12XRARE-tk (positive control), pGL-4.23 (empty vector–negative control), pGL-4.23-*ripply3*-DR1, and pGL-4.23-*ripply3*-DR4.
(TIF)

S1 Table Primers sequences used. Blue sequences indicate the target sequence for the gRNAs.
(PDF)

S2 Table Antibodies used. (PDF)
