# Supplementary figures and images for: HDAC1-mediated repression of the retinoic acid-responsive gene ripply3 promotes second heart field development
Source: PLoS Genet. 2019 May 15;15(5):e1008165. doi: 10.1371/journal.pgen.1008165 (PMC6538190; doi:10.1371/journal.pgen.1008165)

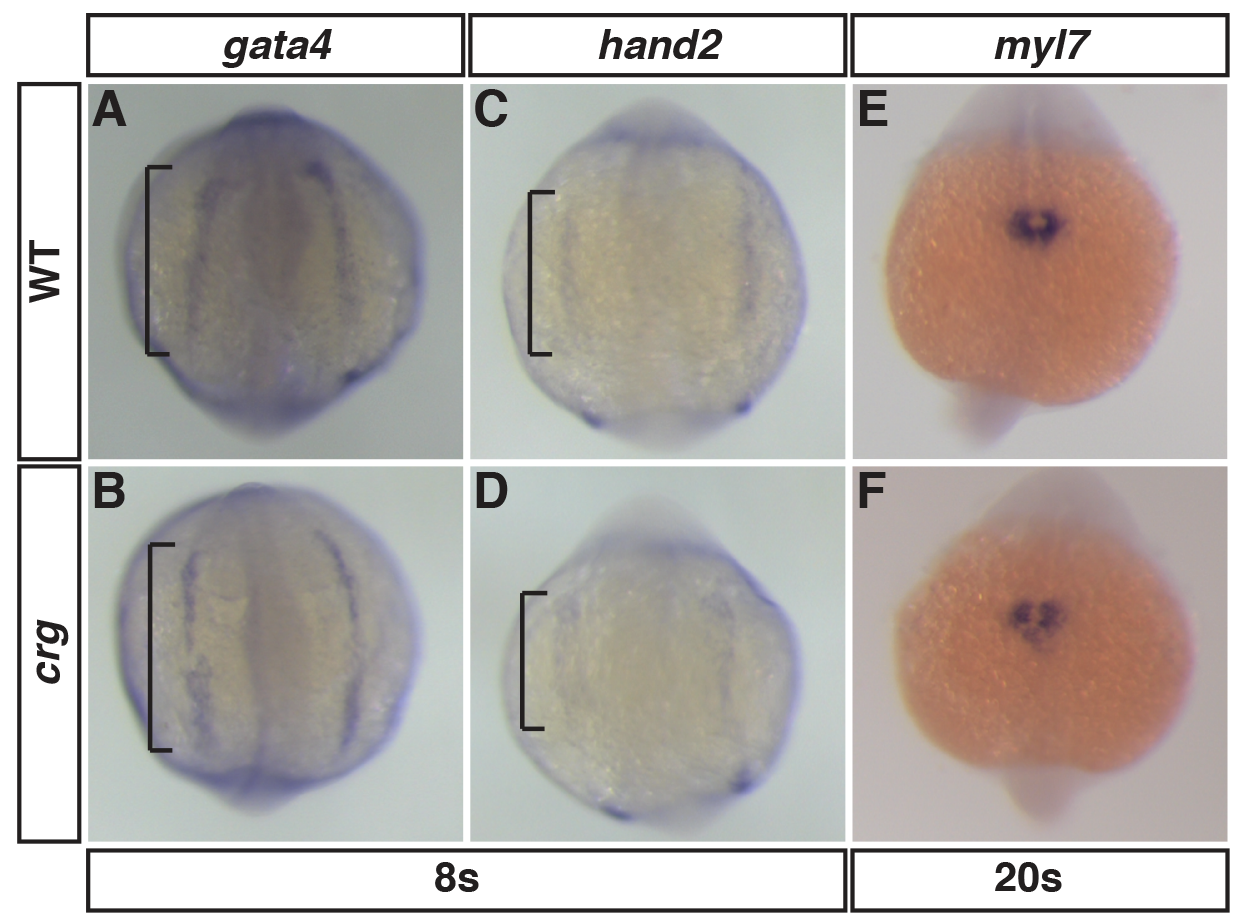

Supplement: S1 Fig — (A-D) ISH for CM specification markers gata4 and hand2 at the 8s stage. n = 5 WT and n = 5 crg mutants embryos examined. (E,F) ISH for the CM differentiation marker myl7 at the 20s stage. n = 10 WT and n = 5 crg mutants embryos examined. Views are dorsal with anterior up. (TIF) [file pgen.1008165.s001.tif]

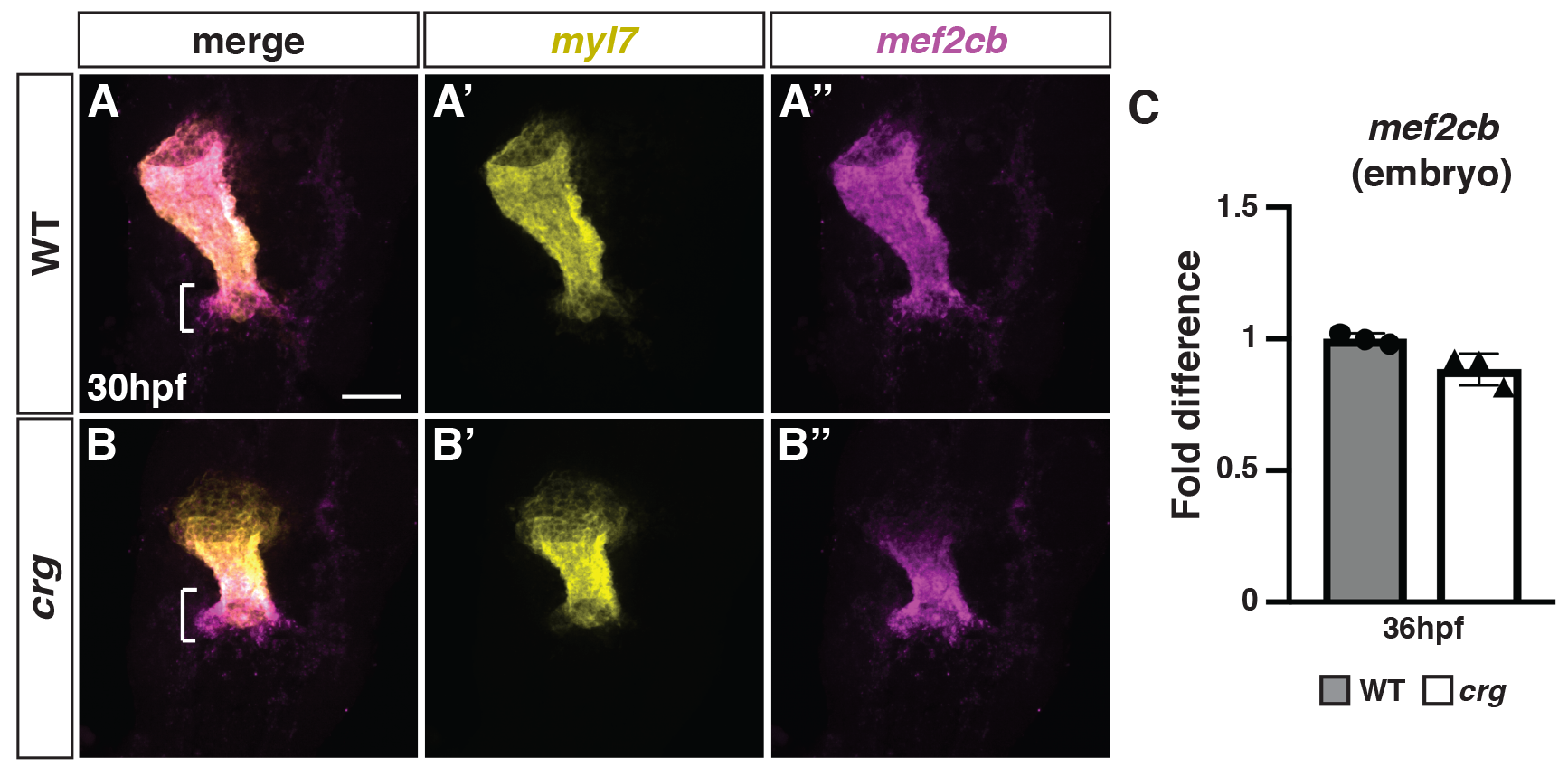

Supplement: S2 Fig — (A-B”) Two-color FISH for myl7 and mef2cb in WT sibling and crg mutant embryos at 30 hpf. Brackets in A and B indicate mef2cb at the arterial pole of in WT and crg mutant hearts, respectively. n = 5 WT and n = 5 crg mutants embryos examined. (C) RT-qPCR for the SHF marker mef2cb from embryos at 36 hpf. (TIF) [file pgen.1008165.s002.tif]

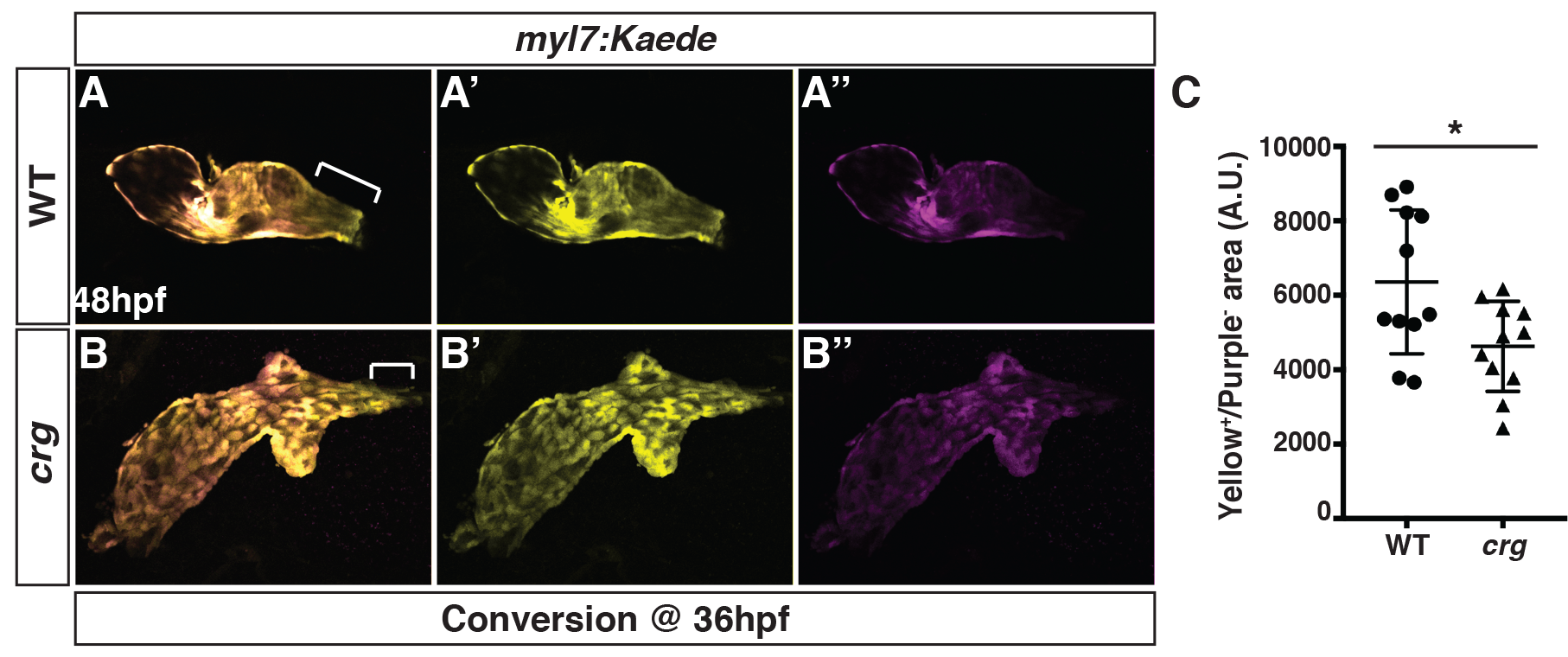

Supplement: S3 Fig — (A-B”) Images of hearts from photoconverted WT sibling and crg mutant myl7:Kaede embryos at 48 hpf following photoconversion at 36 hpf. The arterial poles (brackets) are to the right. (C) Quantification of the area of later-differentiating VCs (Yellow+/Purple-). (n = 11 for WT and crg mutants). (TIF) [file pgen.1008165.s003.tif]

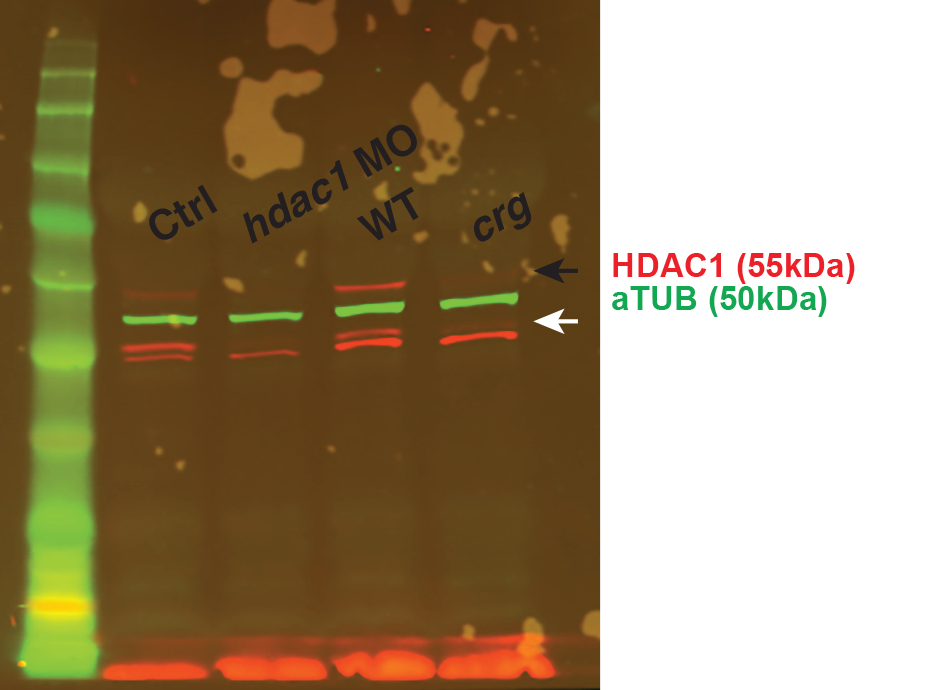

Supplement: S4 Fig — Both the hdac1 MO and crg mutants show a loss of the predicted WT Hdac1 proteins (black arrow). We do not observe the smaller protein with a 32 bp deletion predicted from the transcript analysis in RNA-seq, suggesting that this protein is not generated in the crg mutants. Since the antibody used for Western was generated to the C-terminal, it would not recognize the severely truncate protein, if that were made in crg mutants. However, a lower band (white arrow) recognized by the antibody, which is potentially a smaller Hdac1 isoform that is present in the WT/Ctrl samples, is also lost in the Hdac1-depleted and crg mutant embryos. (TIF) [file pgen.1008165.s004.tif]

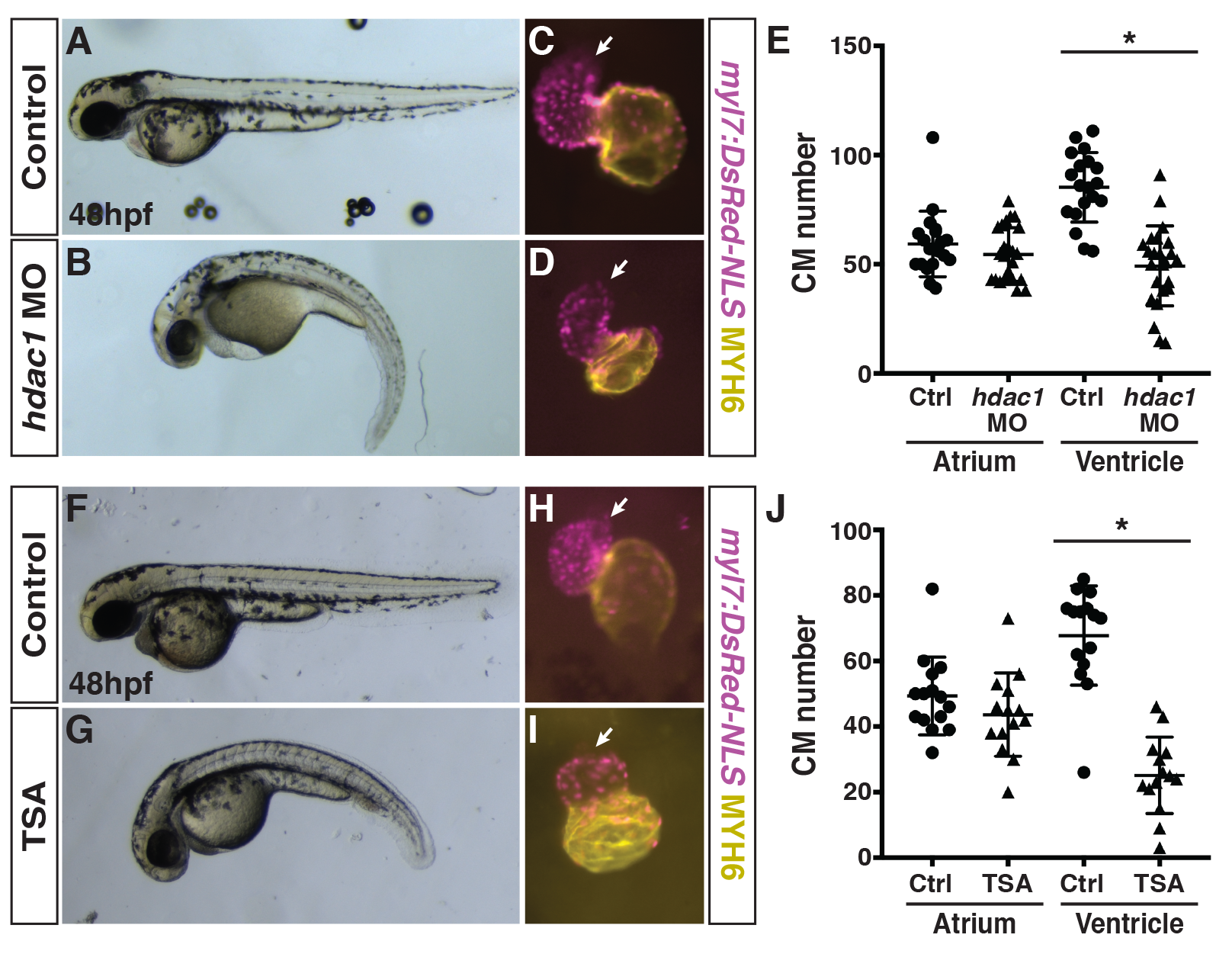

Supplement: S5 Fig — (A,B) Control and Hdac1-depleted embryos at 48 hpf. Lateral views with anterior to the left. (C,D) Hearts from control and Hdac1-depleted myl7:NLS-DsRed2 embryos at 48 hpf. Frontal views. Purple alone indicates ventricle. Yellow indicates atrium. Arrows indicate arterial pole of the ventricle. (E) Quantification of CMs in the atria and ventricles of control and Hdac1-depleted myl7:NLS-DsRed2 embryos at 48 hpf (n = 19 for control and Hdac1-depleted embryos). (F,G) Control and TSA-treated embryos at 48 hpf. Lateral views with anterior to the left. (H,I) Hearts from control and TSA-treated myl7:NLS-DsRed2 embryos at 48 hpf. Frontal views. Purple alone indicates ventricle. Yellow indicates atrium. Arrows indicate arterial pole of the ventricle. (J) Quantification of CMs in the atria and ventricles of control and TSA-treated myl7:NLS-DsRed2 embryos at 48 hpf (n = 14 for both control and TSA-treated embryos). (TIF) [file pgen.1008165.s005.tif]

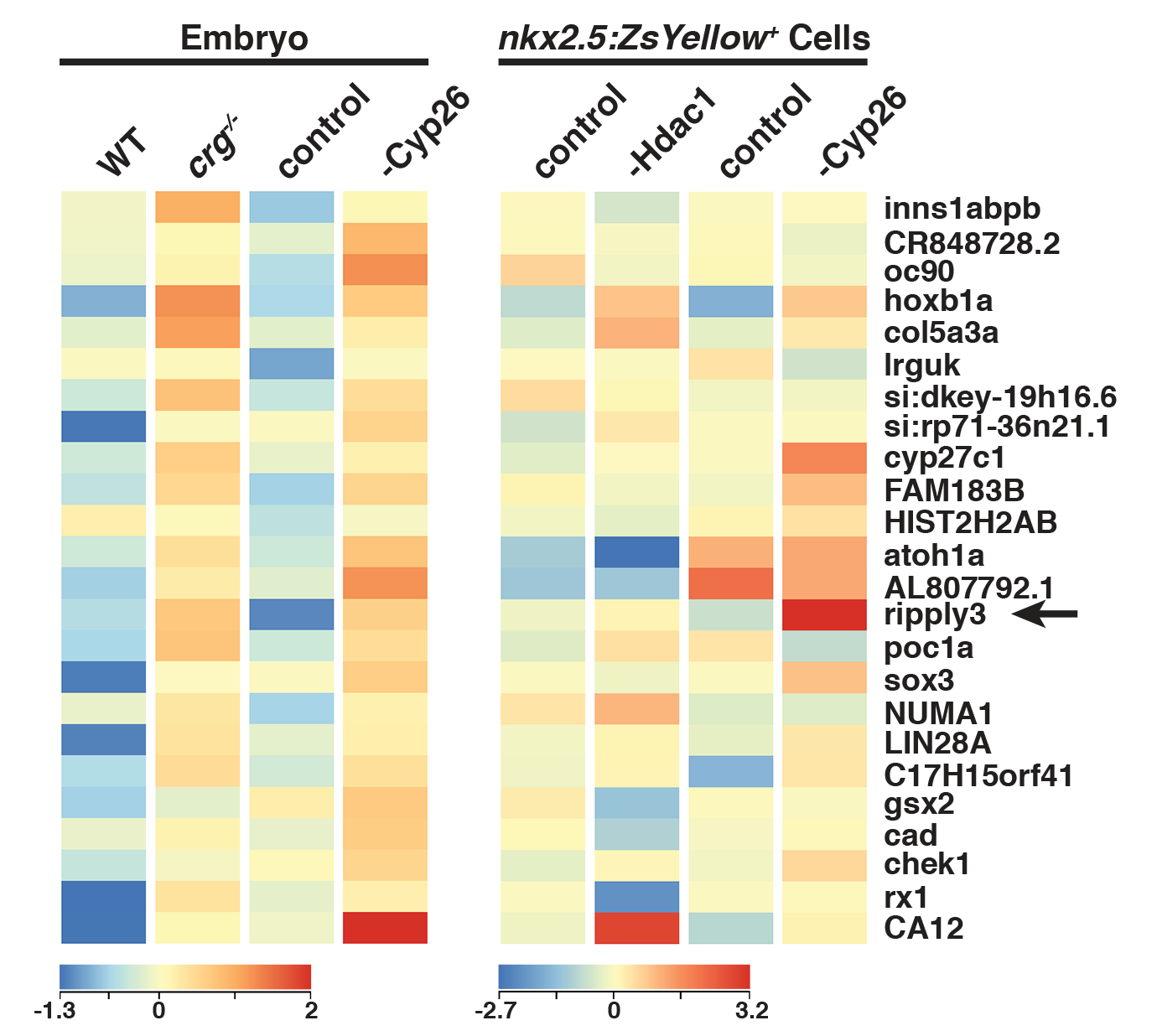

Supplement: S6 Fig — Heat-maps of genes found to have increased expression from RNA-seq of Hdac1- and Cyp26-depleted embryos at 48 hpf and sorted nkx2.5:ZsYellow+ cells at 33 hpf. Scale represents fold-change (log2) of normalized values after DE-seq analysis in Strand NGS. Genes represented showed similar trend of increased expression. (TIF) [file pgen.1008165.s006.tif]

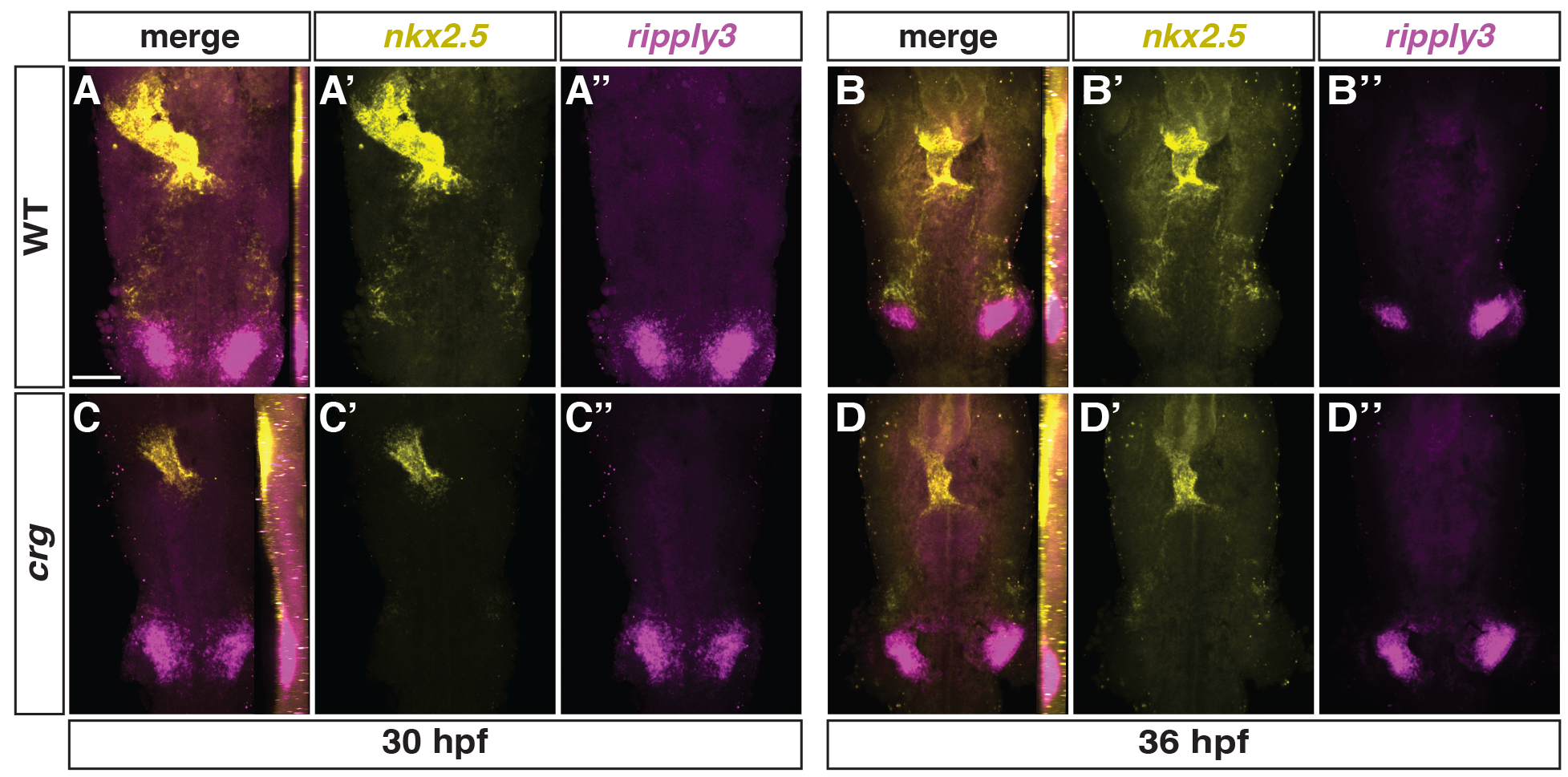

Supplement: S7 Fig — (A-D”) Confocal images of two-color FISH for nkx2.5 and ripply3 in WT and crg mutant embryos at 30 and 36 hpf. Images are dorsal views with anterior up. Insets in A-D indicate lateral views of the confocal images. n = 10 WT and n = 9 crg mutants embryos for 30 hpf and n = 4 WT and n = 4 crg mutants embryos for 36 hpf examined. Scale bar is 100 μm. (TIF) [file pgen.1008165.s007.tif]

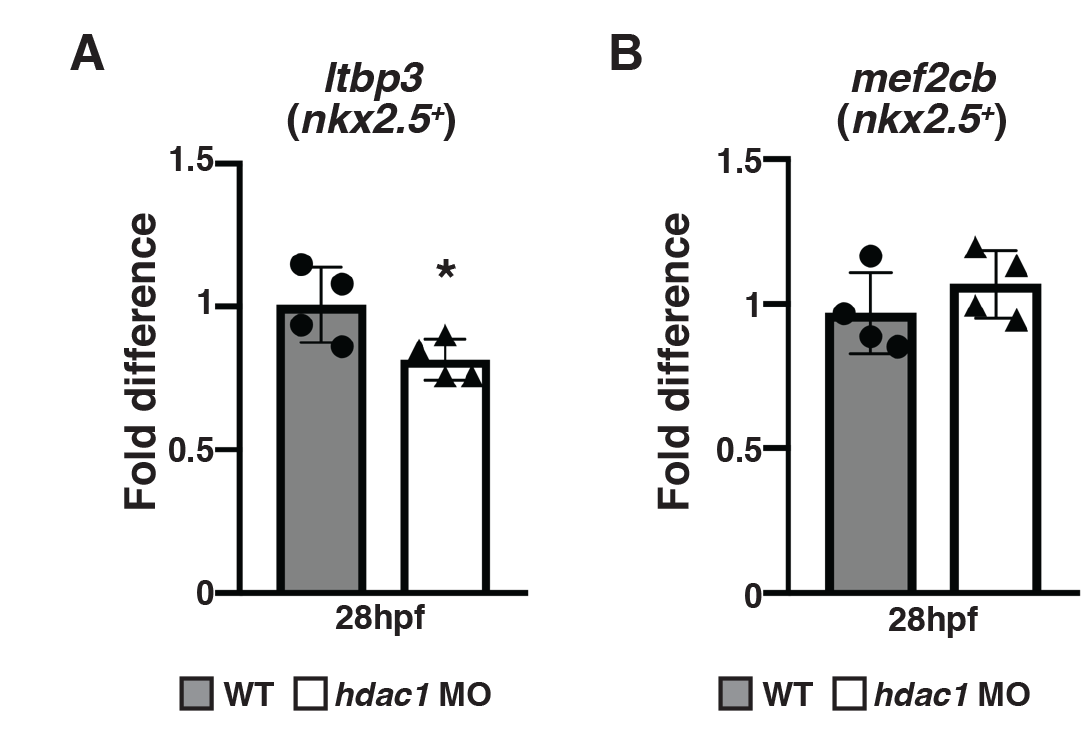

Supplement: S8 Fig — (A) RT-qPCR for ltbp3 from sorted nkx2.5+ cells at 28 hpf. (B) RT-qPCR for mef2cb from sorted nkx2.5+ cells at 28 hpf. (TIF) [file pgen.1008165.s008.tif]

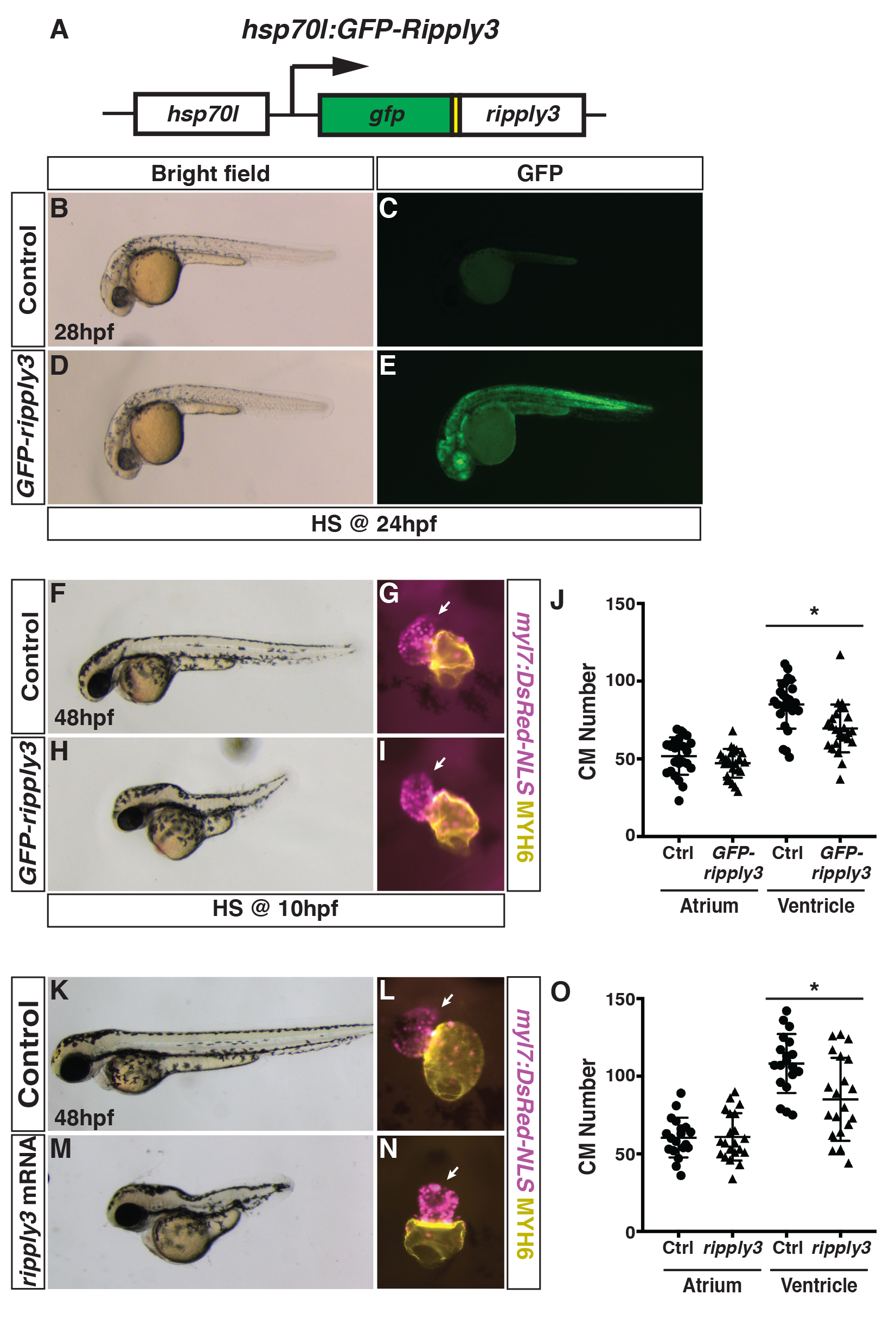

Supplement: S9 Fig — (A) Schematic of the heat-shock inducible hsp70l:GFP-ripply3 transgene. (B-E) Heat-shock induction of GFP-ripply3 at 24 hpf. Control embryos are heat-shocked non-transgenic siblings. (F,H) Control and hsp70l:GFP-ripply3 embryos at 48 hpf following heat-shock at 10 hpf. Lateral views with anterior to the left. (G,I) Hearts from control and hsp70l:GFP-ripply3; myl7:NLS-DsRed2 embryos at 48 hpf following heat-shock at 10 hpf. Frontal views. Purple alone indicates ventricle. Yellow indicates atrium. Arrows indicate arterial pole of the ventricle. (J) Quantification of CMs from control and hsp70l:GFP-ripply3; myl7:NLS-DsRed2 embryos at 48 hpf following heat-shock at 10 hpf (n = 25 for control and GFP-ripply3+). (K,M) Control and ripply3 mRNA-injected embryos at 48 hpf. Lateral views with anterior to the left. (L,N) Hearts from control and ripply3 mRNA-injected myl7:NLS-DsRed2 embryos at 48 hpf. Frontal views. Purple alone indicates ventricle. Yellow indicates atrium. Arrows indicate arterial pole of the ventricle. (O) Quantification of CMs from control and ripply3 mRNA-injected myl7:NLS-DsRed2 embryos at 48 hpf (n = 21 for control and ripply3 mRNA-injected embryos). (TIF) [file pgen.1008165.s009.tif]

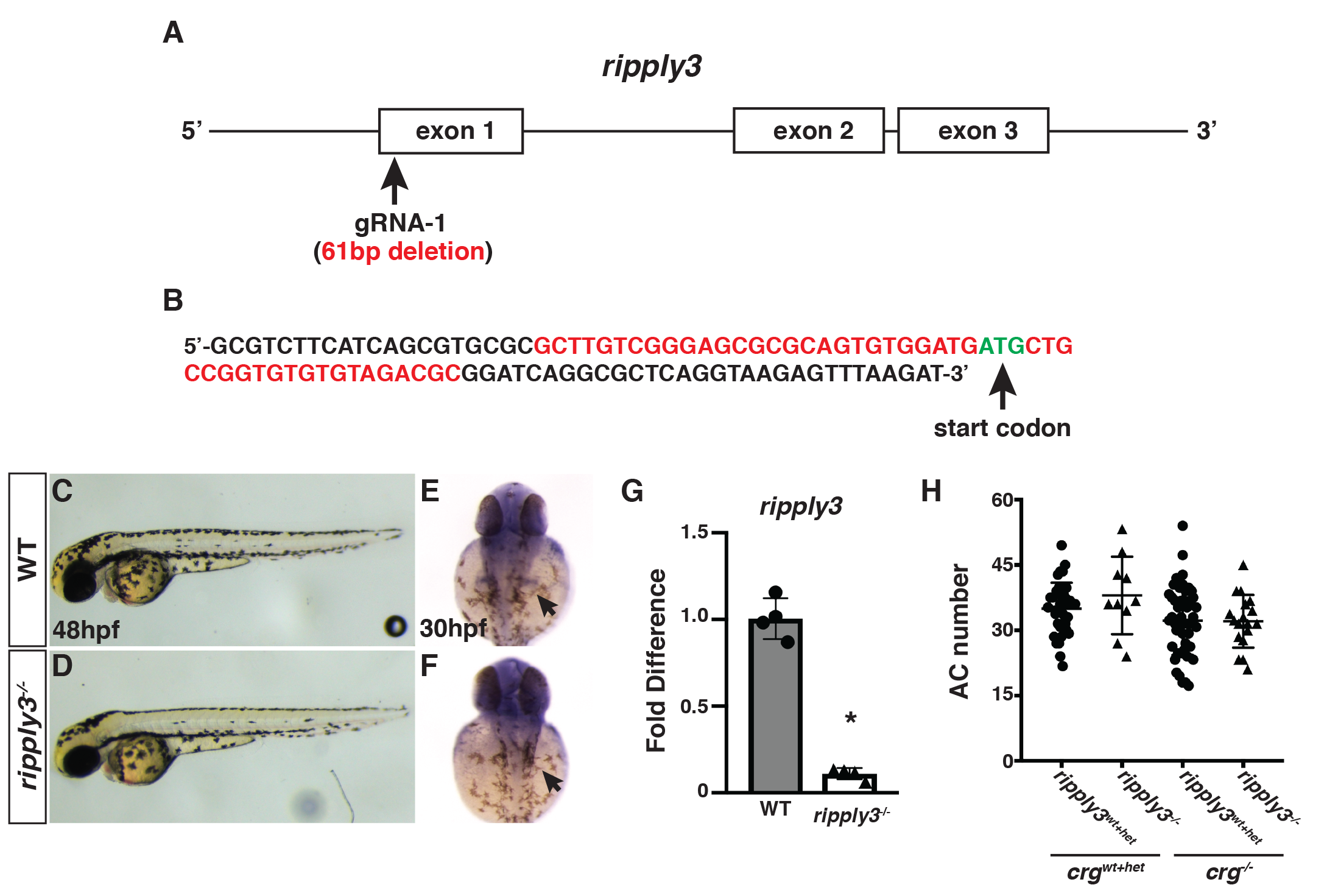

Supplement: S10 Fig — (A) Schematic of the ripply3 exons and the gRNA targeting exon of the ripply3 gene. (B) The ripply3 mutant allele used deletes 61bp including the start codon. (C,D) WT sibling and ripply3 mutants at 48 hpf. (E,F) Ripply3 transcripts are not detectable in ripply3 mutants at 30 hpf. Arrows indicate posterior pharyngeal region where ripply3 is expressed in WT embryos. Views are dorsal with anterior up. (G) RT-qPCR for ripply3 in ripply3 mutants at 36 hpf indicates the transcripts are essentially undetectable. Primers used do not bind within the deleted region. (H) Quantification of atrial CMs in crgwt+het; ripply3wt+het, crgwt+het; ripply3-/-, crg-/-; ripply3wt+het, and crg-/-; ripply3-/- embryos at 48 hpf (n = 37 for crgwt+het; ripply3wt+het, n = 10 for crgwt+het; ripply3-/-, n = 48 for crg-/-; ripply3wt+het, n = 18 for crg-/-; ripply3-/-). (TIF) [file pgen.1008165.s010.tif]

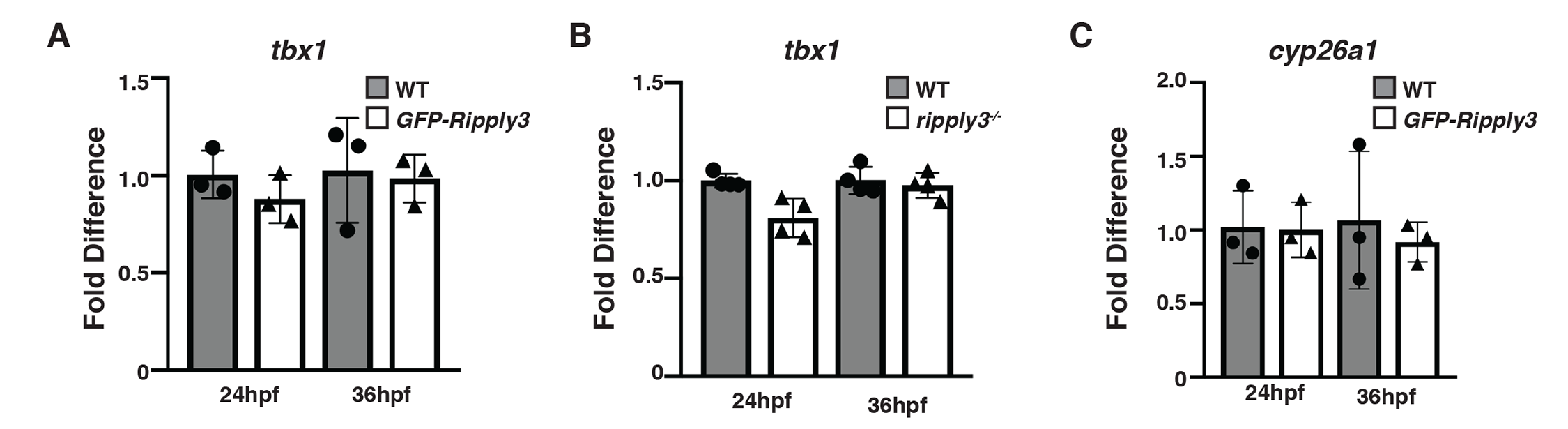

Supplement: S11 Fig — (A-C) RT-qPCR for tbx1 and cyp26a1 from embryos at 24 and 36 hpf following GFP-Ripply3 induction at the 20s stage and ripply3 mutants. (TIF) [file pgen.1008165.s011.tif]

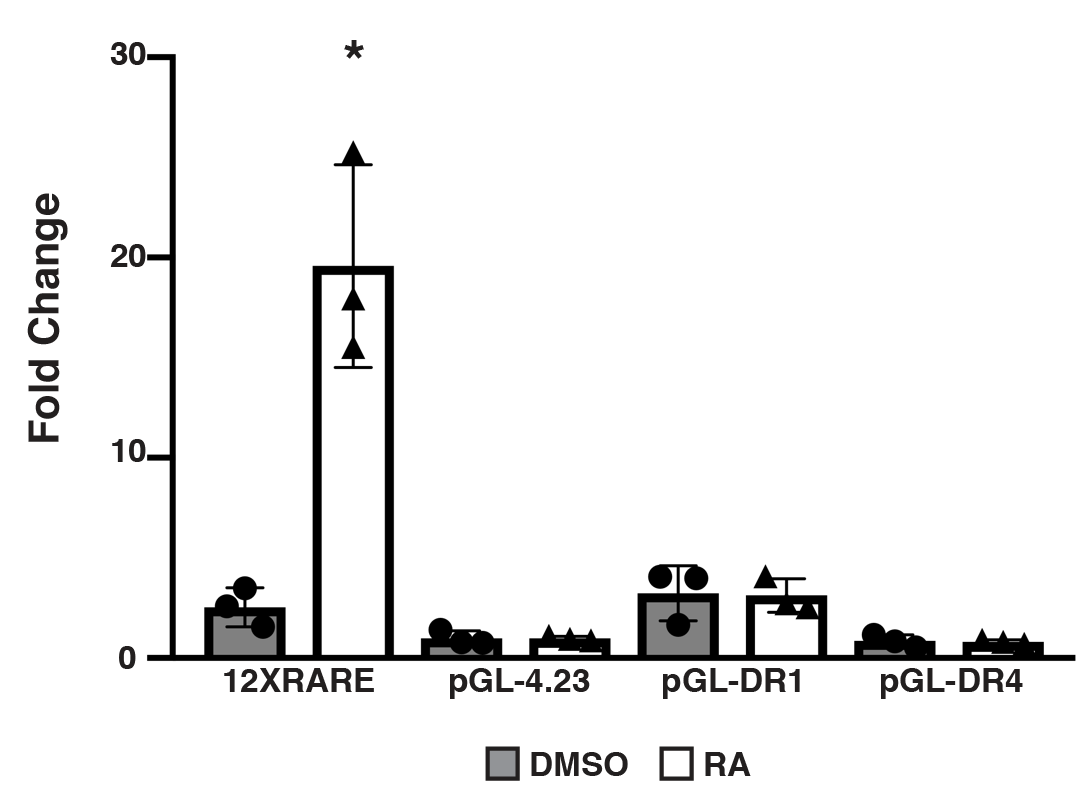

Supplement: S12 Fig — Dual luciferase assays in HEK293 cells for the ripply3 DR1 and DR4 sites. pGL3-12XRARE-tk (positive control), pGL-4.23 (empty vector–negative control), pGL-4.23-ripply3-DR1, and pGL-4.23-ripply3-DR4. (TIF) [file pgen.1008165.s012.tif]
