# Supplementary material for: HDAC1-mediated repression of the retinoic acid-responsive gene ripply3 promotes second heart field development
Source: PLoS Genet. 2019 May 15;15(5):e1008165. doi: 10.1371/journal.pgen.1008165 (PMC6538190; doi:10.1371/journal.pgen.1008165)
Supplement: S1 Table — Blue sequences indicate the target sequence for the gRNAs. (PDF) [file pgen.1008165.s013.pdf]

S1 Table. Primers sequences used.

Application	Primer Name	Sequence
**cloning**	ripply3-linker-f1	GAGAGCTTGGGCGACCTCACCCTGCCGGTGTGTGTAGACG
	ripply3-5long-f1	ATGCTGCCGGTGTGTGTAGACGCGGATCAGGCGCTCAGATGTTCTGTGCTATGGAGACCG
	attB1-gfp-f1	GGGGACAAGTTTGTACAAAAAAGCAGGCTTTCACCATGGTGAGCAAGGGCGAGGAGCTG
	GFP-linker-R	GGTGAGGTCGCCCAAGCTCTCCTTGTACAGCTCGTCCAT
	ripply3-attB2-R1	GGGGACCACTTTGTACAAGAAAGCTGGGTTTCACGGCCTCCAGAGTCTGCAGCG
	ripply3_dr1_f1	GTACGGGGATCTCCTGCATG
	ripply3_dr1_r1	TCGTGTTTCCTCACCTTGGG
	ripply3_dr4_f1	CAAGGCTGCTGAACAAACCC
	ripply3_dr4_r1	CCAGTGAGGAACCGTGAAGG
**genotyping**	crg-HphI-F2	GTCCTATGAAGCCATATTCAAAGGTG
	crg-g-R1	GACCTGAAGAACCCACAAAG
	ripply3-t1-f1	CGACAATGCAATGTTCAACTTA
	ripply3-t2-r1	ACGATAACTGGTCATTTTTGGG
**F0 CRISPR genotyping**	ripply3-DR1-t1-f1	TTACATCTCAAACCAAACGCAG
	ripply3-DR1-t2-r1	GCTTCCACGAGATCTACGAGAC
	ripply3-DR4-t3-f1	ACTGCTTCAGCAGGAGGTAAAG
	ripply3-DR4-t4-r1	TTCCACAGAACCCTAACCCTAA
	ripply3_ctrl_t3_f1	ATTTGCAGAAAAGGGAAAACTG
	ripply3_ctrl_t4_r1	TGGTCATCGTTGGTAAACTCTG
**gRNA**	ripply3-t1	GCGTAATACGACTCACTATAGGTGTGTGTAGACGCGGATCGTTTTAGAGCTAGAAATAGC
	ripply3-t2	GCGTAATACGACTCACTATAGGTCTGTGCTATGGAGACCGGTTTTAGAGCTAGAAATAGC
	Cas9 Guide-R	AAAGCACCGACTCGGTGCCACTTT
	ripply3-DR1-t1	GCGTAATACGACTCACTATAGGGTCTCTGAAACCATGCAGGTTTTAGAGCTAGAAATAGC
	ripply3-DR1-t2	GCGTAATACGACTCACTATAGGAGAGCGAGTCCAAAGTGTGTTTTAGAGCTAGAAATAGC
	ripply3-DR4-t3	GCGTAATACGACTCACTATAGGCCACTGAAGCCCTGTGGAGTTTTAGAGCTAGAAATAGC
	ripply3-DR4-t4	GCGTAATACGACTCACTATAGGCTATGTGCAGTTGATTACGTTTTAGAGCTAGAAATAGC
	ripply3-ctrl-t3	GCGTAATACGACTCACTATAGGAGTCAGGAAAAACTCCTGGTTTTAGAGCTAGAAATAGC
	ripply3-ctrl-t4	GCGTAATACGACTCACTATAGGAACTAGCCACATTACACAGTTTTAGAGCTAGAAATAGC
**EMSA**	rare 2_dr5	CAGGTTCACCGAAAGTTCAAGTAAGGTGAAATGCAGGTCACAAGACATTCGCGCAGATC
	ripply3_dr1	CATTCCTGAGCTGACCTCTGACCTAGACATTCGCGCAGATC
	ripply3_dr4	TTCTGCAGGGAGTTCCTCAGGGGTCAAGACATTCGCGCAGATC
**RT-qPCR**	ripply3-RT-F1	GATGTTCTGTGCTATGGAGACCG
	ripply3-RT-R1	AGCGTGTGTTTGCGTGTTCC
**CHIP-qPCR**	ripply3_DR1_f2	CCAAAGTGTAGGAGGACGGG
	ripply3_DR1_r2	AGAAGTGCGTGTGATGGGGG
	ripply3_DR4_f1	CAGGATGAAGACCTTTCCACAG
	ripply3_DR4_r1	CCCTTGAACACGATACAGCG
**mapping**	z11872f	TGTGGAGGAAGAAGGAAACG
	z11872r	ATGTGGGAGTTTTGCACCTC
	z65805f	CCCATCTGCCCAAGTCTAT
	z65805r	GAGAAACAGGAGGAAGAAAGCA
	z7235f	TGATTCAAAGCAGAGGCTGA
	z7235r	TTCAAGCACGGATTTGAAAA
	z10002f	CGACGGAGCTTACACAACAA
	z10002r	TGATGTCATTGCTCAAAGGG
