# Supplementary material for: HDAC1-mediated repression of the retinoic acid-responsive gene ripply3 promotes second heart field development
Source: PLoS Genet. 2019 May 15;15(5):e1008165. doi: 10.1371/journal.pgen.1008165 (PMC6538190; doi:10.1371/journal.pgen.1008165)
Supplement: S2 Table — (PDF) [file pgen.1008165.s014.pdf]

S2 Table. Antibodies used.

	Antibody	Supplier	Product	Procedure	Dilution
**Primary**	rabbit anti-zebrafish Hdac1	Gene Tex	124499	Western Blot	1:1000
	mouse anti-Alpha-Tubulin	Sigma	6199	Western Blot	1:1000
	rabbit anti-DsRed2	Clontech	632496	Immunostaining	1:1000
	mouse anti-MF20	DSHB	MF20 (supernatant)	Immunostaining	1:10
	mouse anti-S46	DSHB	S46 (supernatant)	Immunostaining	1:10
	rabbit anti-zebrafish NKX2.5	Gene Tex	128357	Immunostaining	1:250
	mouse anti-Histone H3	Abcam	ab14955	Immunostaining	1:500
	mouse anti-c-myc	Biolegend	626802	EMSA	1:1000
	rabbit anti-zebrafish Hdac1	Abcam	ab41407	ChIP	1:100
	rabbit anti-GFP	Abcam	ab290	ChIP	1:100
	rabbit anti-H3K27ac	Abcam	ab4729	ChIP	1:100
	rabbit anti-H3K27me3	Millipore	7449	ChIP	1:100
	sheep anti-Fluorescein-POD	Sigma-Aldrich	11426346910	FISH	1:50
	sheep anti-DIG-POD	Sigma-Aldrich	11207733910	FISH	1:50
	rabbit anti-zebrafish Elastin b	YenZym	N/A	Immunostaining	1:250
	rabbit anti-Zebrafish Vmhc	YenZym	N/A	Immunostaining	1:250
**Secondary**	donkey anti-Mouse IgG (H + L)	Licor	925-32212	Western Blot	1:10000
	donkey anti-Rabbit IgG (H + L)	Licor	925-68023	Western Blot	1:10000
	goat anti-mouse IgG1 TRITC	Southern Biotech	1070-03	Immunostaining	1:100
	goat anti-mouse IgG1 FITC	Southern Biotech	1070–02	Immunostaining	1:100
	goat anti-rabbit IgG-TRITC	Southern Biotech	4050–03	Immunostaining	1:100
	goat Anti-Rabbit IgG-FITC	Southern Biotech	4050-02	Immunostaining	1:100
	goat anti-mouse IgG1-DyLight 405	BioLegend	409109	Immunostaining	1:100
	goat anti-mouse IgG2b TRITC	Southern Biotech	1090–03	Immunostaining	1:100
	goat anti-chicken IgG-FITC	Southern Biotech	6100–02	Immunostaining	1:100
